# Phosphorylation of cell cycle and apoptosis regulatory protein-1 by stress activated protein kinase P38γ is a novel mechanism of apoptosis signaling by genotoxic chemotherapy

**DOI:** 10.3389/fonc.2024.1376666

**Published:** 2024-05-02

**Authors:** Jaganathan Venkatesh, Magesh Muthu, Indulekha Singaravelu, Vino T. Cheriyan, Sreeja C. Sekhar, Nuwan C. P. N. Acharige, Edi Levi, Hadeel Assad, Mary Kay H. Pflum, Arun K. Rishi

**Affiliations:** ^1^ John D. Dingell V.A. Medical Center, Wayne State University, Detroit, MI, United States; ^2^ Karmanos Cancer Institute, Wayne State University, Detroit, MI, United States; ^3^ Department of Oncology, Wayne State University, Detroit, MI, United States; ^4^ Department of Chemistry, Wayne State University, Detroit, MI, United States; ^5^ Department of Pathology, Wayne State University, Detroit, MI, United States

**Keywords:** genotoxic chemotherapy, CCAR1/CARP-1, phosphorylation, P38γ, breast cancer

## Abstract

CARP-1, a perinuclear phospho-protein, regulates cell survival and apoptosis signaling induced by genotoxic drugs. However, kinase(s) phosphorylating CARP-1 and down-stream signal transduction events remain unclear. Here we find that CARP-1 Serine (S)^626^ and Threonine (T)^627^ substitution to Alanines (AA) inhibits genotoxic drug-induced apoptosis. CARP-1 T^627^ is followed by a Proline (P), and this TP motif is conserved in vertebrates. Based on these findings, we generated affinity-purified, anti-phospho-CARP-1 T^627^ rabbit polyclonal antibodies, and utilized them to elucidate chemotherapy-activated, CARP-1-dependent cell growth signaling mechanisms. Our kinase profiling studies revealed that MAPKs/SAPKs phosphorylated CARP-1 T^627^. We then UV cross-linked protein extracts from Adriamycin-treated HeLa cervical cancer cells with a CARP-1 (614–638) peptide, and conducted liquid chromatography-tandem mass spectrometry (LC-MS/MS) analyses of the peptide-bound protein complexes. This experiment revealed SAPK p38γ interaction with CARP-1 (614–638) peptide. Our studies further established that SAPK p38γ, but not other MAPKs, phosphorylates CARP-1 T^627^ in cancer cells treated with genotoxic drugs. Loss of p38γ abrogates CARP-1 T^627^ phosphorylation, and results in enhanced survival of breast cancer cells by genotoxic drugs. CARP-1 T^627^ phosphorylation was also noted in breast tumors from patients treated with radiation or endocrine therapies. We conclude that genotoxic drugs activate p38γ-dependent CARP-1 T^627^ phosphorylation to inhibit cell growth.

## Introduction

CARP-1/CCAR1 is biphasic transducer of cell growth, survival, and apoptosis signaling (1). CARP-1 functions to transduce growth and survival signaling by steroid/thyroid receptor superfamily members, Wnt/β-catenin, notch receptors, APC/C E3 ligase, and NF-κB, as well as regulates endocrine differentiation (2–8). CARP-1 also transduces apoptosis signaling by a variety of genotoxic chemotherapies such as Adriamycin (Doxorubicin), Etoposide, Cisplatin, and CFM experimental compounds, as well as by the EGFR TKIs gefitinib, Erlotinib, and Osimertinib (7–16).

CARP-1 is a ubiquitous, ~130kDa protein (17) that has homologs in vertebrates, *Apis millifera*, and the worm *Caenorhabditis elegans*. CARP-1 promoter methylation as well as signaling by protein kinase A (PKA) also regulate CARP-1 expression and function respectively (18, 19). Although the EGF as well as the ATM kinase signaling target specific serine residues of CARP-1 (17, 20, 21), the precise role(s) and kinase(s) of CARP-1 serine phosphorylation remain unclear. High through-put phospho-proteomic analyses at the phosphosite plus portal (22) indicate a number of potential phosphorylation, ubiquitination, and acetylation sites in CARP-1 protein. Although highest number of hits are indicated for T^627^ phosphorylation of human CARP-1 protein, the precise identity of the kinase and significance of CARP-1 T^627^ phosphorylation and down-stream signaling remain unknown.

Here we report that expression of CARP-1 protein having substitutions of S^626^ and T^627^ amino acids to Alanines results in abrogation of cell growth suppression by DNA damage-inducing agents Adriamycin (ADR) or CFM compounds that involves reduced apoptosis. Our *in vitro* kinase profiling and mutagenesis studies together with LC-MS/MS analyses of the CARP-1 peptide-bound protein complexes revealed that SAPK p38γ interacts with and phosphorylates CARP-1 T^627^. Genotoxic stress-induced CARP-1 T^627^ phosphorylation regulates SAPK p38γ-dependent cell growth and survival.

## Materials and methods

### Materials

Cell culture media (DMEM, DMEM Glutamax, EMEM, Opti-MEM) and antibiotics (penicillin and streptomycin) were purchased from Invitrogen Co. (Carlsbad, CA). DMSO and Fetal bovine serum (FBS) were purchased from Fisher Scientific (Fair Lawn, NJ) and Denville Scientific Inc. (Metuchen, NJ), respectively. Protein Assay Kit was purchased from Bio-Rad Laboratories (Hercules, CA) while Chemi-luminescence Reagent was purchased from Amersham Biosciences (Piscataway, NJ). Structure and synthesis of experimental compounds CARP-1 Functional Mimetic (CFM)-4, -5, and -16 have been described previously (14). Clinical grade Adriamycin, Cisplatin, 5-fluouracil, Gemcitabine, and Paclitaxel were obtained from the Karmanos Cancer Institute Pharmacy, Wayne State University, Detroit, MI. 5-dimethyltiazol-2-yl-2.5-diphenyl-tetrazolium bromide (MTT) was purchased from Sigma Chemical Co, St. Louis, MO. The affinity purified, anti-CARP-1 (α1 and α2) polyclonal antibodies have been described (9). The affinity purified, anti-phospho-CARP-1 T^627^ rabbit polyclonal antibodies were generated and characterized on a fee-for-service basis by Kaneka Eurogentec, Seraing-Belgium. Additional, commercially available antibodies utilized in the study are listed in **Supplementary Table S1**.

### Recombinant plasmid constructs, cell lines and cell culture

The plasmids for expression of various proteins were either procured from Addgene or generated in this report and are listed in **Supplementary Table S2**. All the recombinant plasmids were sequenced to confirm the accuracy and validity of various inserts/epitopes.

Routine maintenance and culture of different cell lines was carried out as described in reports listed for respective cell line in **Supplementary Table S3**. MDA-MB-468 cells having CRISPR-based p38γ knock-out were generated and characterized on a fee-for-service basis by Biocytogen Corp., Wakefield, MA. All the cell culture media were also supplemented with 10%FBS, 100units/ml of penicillin, and 100μg/ml of streptomycin, and the cells were maintained at 37°C and 5% CO_2_. Generation, characterization, and culture of drug (ADR or Cisplatin)-resistant human TNBC MDA-MB-468 and MDA-MB-231 cells, ADR-resistant murine 4T1 cells, as well as MDA-MB-468 cells expressing reduced CARP-1 have been detailed before (9, 14). For cell growth and MTT studies, the cells were cultured in fresh media with 5%-10% FBS prior to their treatments with various agents.

The stable sublines are listed in **Supplementary Table S4**, and were generated by transfecting various plasmids into the MDA-MB-468 and Hela cells followed by selection in the presence of 800μg/ml neomycin using described methods (8, 9, 14). The cell lysates from wild-type, untransfected cells, neomycin-resistant pools, or individual sublines were then subjected to IP and WB analyses as below.

### Cell viability, immunoprecipitation, luciferase and western blot assays

Logarithmically growing cells were seeded in 96 well plate (500-1000 cells/well) and then were either untreated (control) or treated with various agents for noted times. At the end of treatment, cells were incubated with MTT reagent (0.5mg/ml) for 2-4 hours at 37°C, followed by addition of DMSO to solubilize formazan. The plate was read at 570nm in a plate reader, and histograms indicating levels of cell viability were generated by plotting the net absorbances as described (8, 9, 14). For western blot and immunoprecipitation experiments, logarithmically growing cells were either untreated or treated with different agents for various time periods followed by lysis of cells to prepare protein extracts. Western blotting was carried out by electrophoresis of 50-100µg of respective protein lysate on 8-12% SDS-PAGE. For immuno-precipitation (IP), the cell lysates (1mg/sample) were first incubated with appropriate antibodies, and the protein complexes were analyzed by SDS-PAGE as above. The proteins on gels were then transferred to supported nitrocellulose membranes followed by hybridization of membranes with primary antibodies essentially as described (8, 9, 14).

### Immunohistochemical staining of human breast cancer tumor microarrays

Human breast tumor TMAs were obtained from Dr. George Sandusky, Indiana University Simon Cancer Center Tissue Bank Biorepository, Indianapolis, IN. TMAs were derived from 14 blocks with ~45 patients/block. A total of 504 tumor specimens with non-identifiable patient treatment and survival outcome were analyzed. Immunostaining of the TMA slides was carried out with anti-phospho-CARP-1 antibodies (1:1,500 dilution) as well as with total CARP-1 (α2) antibodies essentially following methods described by us before (8, 9, 13, 14, 16). Staining for phospho-CARP was scored by Dr. Edi Levi, Staff Pathologist and Associate Chief of Staff (Research), John D. Dingell VA Medical Center, Detroit, MI. Nuclear staining was arbitrarily assigned as 1 vs 0 with minimum 20% staining as cut off. Images were taken using Zeiss LSM 510 Meta NLO (63X) essentially as described before (11).

### Kinase profiling and K-CLASP experiments

To identify the phosphorylating kinases, we utilized two experimental strategies. In the first approach, a fee-for-service kinase profiling was carried out by SignalChem Lifesciences Corporation, Richmond, BC, Canada. Various protein kinases and their respective substrates were generated in-house at SignalChem using proprietary methods followed by their routine quality control testing of each kinase and its substrate to ensure compliance to acceptable standards. The assay condition for each of the protein kinase were optimized to yield acceptable enzymatic activity and high signal-to-noise ratio. ^33^P-ATP was purchased from PerkinElmer. All other materials were of standard grade. The wild-type CARP-1 peptide (EQDEE EKDDG EAKEI STPTH WSKLD PKTMK KK), and its mutant versions (EQDEE EKDDG EAKEI AAPTH WSKLD PKTMK KK) and (EQDEE EKDDG EAKEI AAPAH WSKLD PKTMK KK) were synthesized and reconstituted in dH_2_O and used in phosphorylation experiments. Protein kinase assays (in duplicate) were performed at 30°C for 30 min in a final volume of 25µl containing 5µl of diluted active protein kinase, 5µl of stock solution of regular substrate or respective CARP-1 peptide, 5µl of assay buffer, 5µl of protein kinase activator or assay buffer, and 5µl of ^33^P-ATP (250µM stock solution, 0.8µCi). The reaction was terminated by spotting 20µl of the reaction mixture onto phosphocellulose P81 plate, followed by washing (x3) for ~15 minutes each in a 1% phosphoric acid solution. The radioactivity on the plate was measured in a scintillation counter. Blank control, which included all the assay components except the addition of the appropriate substrate (replace with equal volume of assay dilution buffer), was set up for each protein kinase. The corrected activity for each protein kinase was determined by removing the blank control value. The corrected activity values for CARP-1 peptides were calculated relative to activity values for normal substrate of respective kinase that were set at 100%.

K-CLASP (Kinase- catalyzed Cross linking and streptavidin purification) method to identify the phosphorylating kinases of a known phosphosite was also used. HeLa cell lysate (500 µg total protein) was combined with protein kinase buffer (10% v/v, New England Biolabs, B6022S). Kinase-catalyzed crosslinking was initiated by adding ATP-ArN_3_ (5 mM; synthesized as previously described; 23) and either N-biotin wild type CARP-1 (614–638) (biotin-EEEKDDGEAKEISTPTHWSKLDPKT, synthesized by ProImmune) or N-biotin mutant CARP-1 (614-638; S^626^,T^627^/AA) (biotin-EEEKDDGEAKEIAAPTHWSKLDPKT, synthesized by ProImmune) peptides (1 mM) in a total volume of 100 µL. The crosslinking reactions were incubated at 31°C for 2 hours with shaking at 300 rpm with or without UV irradiation at 365nm. Excess N-biotin peptide and endogenous biotin were removed using Amicon Ultra-0.5 centrifugal filters (0.5 mL, Millipore, UFC500396). The filtered samples (200 µL) were then incubated with prewashed (200µL of phosphate-binding buffer three times) streptavidin resin (400 µL bead slurry, Genscript) for 10 minutes at room temperature with rotation. The bound resin was collected by centrifuging (500 rcf, 1 min, RT) with subsequent ten washes with phosphate-binding buffer (200µL) and then four washes with water (200 µL). Proteins were eluted by boiling in 2% SDS in water (200 µL) for 8 minutes and desalted using 10% SDS-PAGE in preparation for LC-MS/MS analysis.

For LC-MS/MS, digested peptides were separated by reverse-phase chromatography under acidic conditions (0.1% formic acid) using an EASY nLC-1000 UHPLC system (Thermo). Peptides were next analyzed on a Q-Exactive mass spectrometer (Thermo). MS1 profiling was carried out over a 375-1600 m/z range at a resolution of 70,000. MS2 fragmentation was performed using higher energy collision-induced dissociation (HCD) on the top 15 ions using a 1.6 m/z window and normalized collision energy of 29. Dynamic exclusion was turned on (15 s). MS raw data were processed using MaxQuant (version 1.5.2.8) against a human protein database from UniProt (downloaded 2016.04.07, 20159 entries). Searches included up to 2 missed tryptic cleavages. Mass tolerances for parent ions were 20 ppm for the first search and 4.5 ppm for the second search and 20 ppm for fragment ions. The S5 iodoacetamide derivative of cysteine was specified as a fixed modification. Oxidation of methionine and acetylation of protein N-termini were set as variable modifications. Minimum protein and peptide identification probabilities were set at ≤1% false discovery rate (FDR) as determined by a reversed database search, and proteins required just 1 unique peptide. All other parameters were left at their default settings. A total of 737 proteins were observed (**Supplementary Table S5**). Nonspecifically bead bound proteins were removed by requiring higher intensities in the CARP-1 (614–638) peptide sample with UV light sample compared to without UV light. Among the remaining proteins, possible kinases and associated proteins were selected by generating an enrichment value by dividing the protein intensities from the CARP-1 (614–638) peptide with ATP-ArN_3_ and UV light sample by the CARP-1 (614-638; S^626^,T^627^/AA) with ATP-ArN_3_ and UV light sample from each trial. If the enrichment values of a protein were ≥ 1.2 in at least 3 out of 4 trials, that protein was considered a hit (**Supplementary Table S6**).

### Statistical analyses

The statistical analyses were performed using Prism 6.0 software. The data were expressed as mean ± SEM and analyzed using two-tailed student t-test or one-way ANOVA followed by a *post hoc* test. A p value of <0.05 was considered statistically significant.

## Results

### CARP-1 S^626^,T^627^ regulate signaling by DNA damaging agents

**Figure 1:**
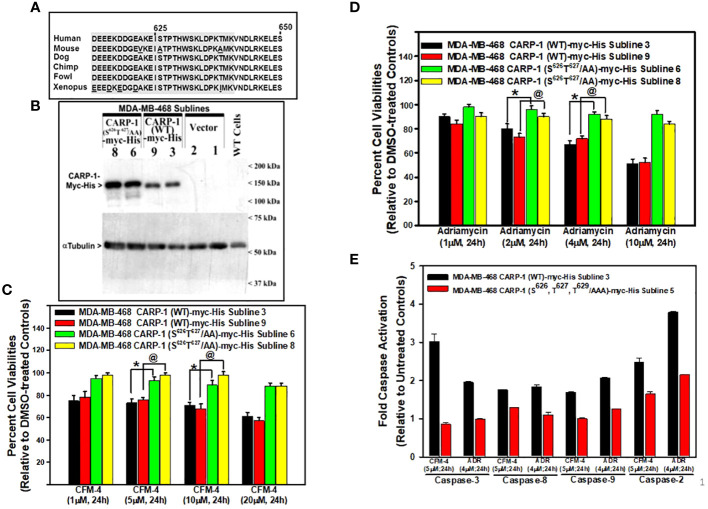
Expression of CARP-1 mutant with substitution of S^626^T^627^/AA abrogates loss of cell viabilities following treatments with DNA damage-inducing agents, in part through reduced activation of pro-apoptotic caspases. **(A)** Homology analysis of amino acids of vertebrate CARP-1 proteins from amino acids 613-650. Amino acids surrounding T^627^ are shaded. **(B)** W.B. analysis of nitrocellulose membrane containing protein lysates derived from untransfected, wild type (WT) HBC cells and stable, neomycin-resistant, HBC sublines expressing vector, myc-His-tagged CARP-1 (WT), or myc-His-tagged CARP-1 (S^626^T^627^/AA) mutant plasmid probed with anti-myc-tag (upper blot) or anti-tubulin (lower blot) antibodies. Arrowheads on the left or right side, respectively, indicate presence of the proteins and molecular weight markers. **(C, D)**, indicated cell lines were treated with DMSO (Control), or with the noted dose and time of indicated agents. Determination of viable/live cells was carried out by MTT assays as detailed in Methods. The bar chart columns represent means of two independent experiments; bars, SE; *, @, p ≤0.001 relative to respective sublines expressing CARP-1 (WT)-myc-His. **(E)**, indicated cell lines were either untreated or treated with noted time and dose of respective agent. Cell lysates were added to the wells of plate that had immobilized fluorogenic substrates of noted caspases. The fluorescence released from the activated caspase-dependent cleavage of respective substrate was detected by plate reader at the excitation and emission wavelengths of 380nm and 460nm, respectively. The columns in bar chart represent means of three independent experiments; bars, SE.

Our prior and on-going studies have indicated that DNA damage-inducing genotoxic agents such as ADR, Cisplatin, or CFM compounds promote apoptosis in part by stimulating levels of CARP-1, phosphorylated SAPKs p38α/β and JNK1/2, cleaved PARP, and activated caspases-3, and -8 (7–16). Knockdown of CARP-1 or pharmacological inhibition of caspase-3 and -8 abrogated apoptosis by ADR, CFM compounds, or EGFR TKIs (7, 9, 11, 14, 16). Moreover, expression of CARP-1 with in-frame deletion of amino acids 600-650 (CARP-1 Δ600-650) also abrogated apoptosis by ADR and CFM compound (11). CARP-1 600-650 region also contains highly conserved, putative consensus TP epitope (T^627^P^628^) for the proline-directed MAPK/SAP kinases (**Figure 1A**), and a high number of hits for potential phosphorylation of T^627^ are also indicated at the phosphosite plus platform (22). CARP-1 S^626^ and T^629^ sites also had a few hits for phosphorylation, albeit these amino acids were also highly conserved in vertebrates with the exception of S^626^ in the rodent (mouse and rat) CARP-1 proteins. Therefore, we first investigated whether CARP-1 S^626^, T^627^, and/or T^629^ residues regulated signaling by DNA damage-inducing agents. We generated and characterized stable, neomycin-resistant human breast cancer (HBC) MDA-MB-468 and cervical cancer Hela cells that express myc-His-tagged CARP-1 protein where S^626^, T^627^, and/or T^629^ were substituted to alanines (CARP-1 S^626^T^627^/AA and CARP-1 S^626^T^627^T^629^/AAA) as detailed in Methods (**Figure 1B**; **Supplementary Figures S1A, C**). ADR or CFM-4 treatments resulted in generally higher viabilities of cells expressing myc-His-CARP-1 S^626^T^627^/AA or myc-His-CARP-1 S^626^T^627^T^629^/AAA when compared with their myc-His-CARP-1 expressing counterparts (**Figures 1C, D**; **Supplementary Figures S1B, D**). Increased viabilities of ADR or CFM-4-treated myc-His-CARP-1 S^626^T^627^T^629^/AAA cells was due in part to diminished expression of caspases-2, -3, -8, and -9 (**Figure 1E**) as well as diminished activation of SAPK JNK1/2 (**Supplementary Figures S2A, B**).

We next investigated the kinases that phosphorylate CARP-1 S^626^, T^627^, and/or T^629^ amino acid(s). In the first instance, we conducted *in vitro* kinase assays utilizing CARP-1 (611–640) wild-type or CARP-1 (611–640) peptides with S^626^T^627^/AA or S^626^T^627^T^629^/AAA substitutions in conjunction with various active serine/threonine protein kinases as detailed in methods. The initial profiling experiment indicated possible phosphorylation of the wild-type but not mutant peptides by ERK1, JNK1, CDK1/Cyclin B1, and CDK1/Cyclin A1 (**Supplementary Table S7**). As CARP-1 T^627^ is followed by a proline, and the TP motif is preferred site of protein phosphorylation by MAPK/SAPKs, a subsequent kinase profiling experiment was performed by utilizing the above peptide substrates in conjunction with various active kinases that included respective MAPK/SAPK isoforms. The data revealed phosphorylation of the wild-type but not mutant peptides by ERK1, ERK2, JNK1, JNK2, JNK3, P38γ, and p38δ but not by p38α or p38β (**Table 1**). Of note is a rather robust phosphorylation of CARP-1 (611–640) wild-type peptide by p38γ. We then investigated whether and to the extent different MAPK/SAPK isoforms function to regulate signaling by DNA damage-inducing agents. We first generated and characterized multiple, stable neomycin-resistant MDA-MB-468 HBC and HeLa cervical cancer sublines that express catalytically-inactive MAPK/SAPK isoforms (ERK1 AEF; JNK1a1 APF; JNK2a2 APF; p38δ AGF; p38γ APF; and p38γ AGF) as described in methods (**Supplementary Figures S3A-C, F, G, K, L**). In addition, we generated and characterized MDA-MB-468 HBC and HeLa cervical cancer sublines that express anti-sense cDNAs of p38δ and p38γ to knock-down the respective kinase proteins (**Supplementary Figures S3D, E, H, I**). MDA-MB-468 HBC and HeLa cervical cancer cells (wild-type, vector transfected, or transfected with catalytically-inactive and antisense cDNAs of above MAPK/SAPK isoforms) were then treated with DNA damage inducing agents ADR, Etoposide, and CFM-4, -5, and -4.16 compounds, followed by determination of cell viabilities by MTT assays as noted in methods. The cells expressing catalytically-inactive JNK1a1, JNK2a2, p38δ and p38γ as well as the sublines with knockdown of p38δ and p38γ were resistant to inhibitory effects of DNA damage-inducing compounds when compared with their wild-type or vector expressing counterparts (**Table 2**; **Supplementary Figures S4B-F**, **S5B-F**). However, the cells expressing catalytically inactive ERK1 were found to be more sensitive to inhibitory effects of DNA damage-inducing compounds when compared with their wild-type or vector expressing counterparts (**Table 2**; **Supplementary Figures S4A**, **S5A**). These data indicate that DNA damage signaling likely activates ERKs to promote cell survival and consequent resistance. The genotoxic agents activate JNKs and p38 SAPKs to promote cell growth inhibition.

**Table 1:** *In vitro* phosphorylation of CARP-1 peptides by various kinases.

Target Kinase	Percent Phosphorylation of CARP-1 Peptide		
	Wild-type	S^626^T^627^/AA	S^626^T^627^T^629^/AAA
JNK1	13	2	3
JNK2	48	2	2
JNK3	46	3	3
ASK2	3	2	3
RIPK2	1	1	2
RIPK5	1	1	2
MEK2	2	2	2
ERK1	32	0	0
ERK2	46	0	0
ERK5	9	1	1
RIPK1	1	1	0
ASK1	7	3	2
p38α	2	0	0
p38β	5	0	0
p38δ	21	0	0
p38γ	63	0	0
MEK1	7	0	1
TAK1	3	3	4
RIPK3	4	4	4

WT EQDEEEKDDGEAKEISTPTHWSKLDPKTMK.

S^626^T^627^/AA EQDEEEKDDGEAKEIAAPTHWSKLDPKTMK.

S^626^T^627^T^629^/AAA EQDEEEKDDGEAKEIAAPAHWSKLDPKTMK.

**Table 2:** Alterations in viabilities of cancer cells expressing kinase-inactive mutants of MAPKs/SAPKs following exposure to DNA damage-inducing agents.

Stable Cell Lines	Catalytically Inactive MAPK/SAPK	MAPK/SAPK knockdown	Cell Viability Increase (↑) or Decrease (↓) Relative to Respective Vector or Wild-type Cells; ND, Not Done				
			Adriamycin	CFM-4	CFM-5	CFM-4.16	Etoposide
MDA-MB-231	P38γ (APF)		↑	↑	ND	↑	↑
		P38γ	↑	↑	ND	↑	↑
HeLa	ERK1 (AEF)		↓	ND	ND	↓	ND
	JNK1a1 (APF)		↑	ND	ND	↑	ND
	JNK2a2 (APF)		↑	↑	↑	ND	ND
		P38δ	ND	ND	ND	ND	ND
		P38γ	↑	↑	ND	ND	↑
	P38γ (APF)/(AGF)		↑	↑	ND	↑	↑
MDA-MB-468	ERK1 (AEF)		↓	ND	ND	↓	ND
	JNK1a1 (APF)		↑	ND	ND	↑	ND
		P38δ	↑	ND	ND	ND	ND
	P38δ (AGF)		↑	ND	ND	ND	ND
	JNK2a2 (APF)		↑	↑	↑	ND	ND
		P38γ	ND	ND	ND	ND	ND
	P38γ (APF)/(AGF)		↑	ND	ND	↑	ND

ND, Not Done.

**Figure 2:**
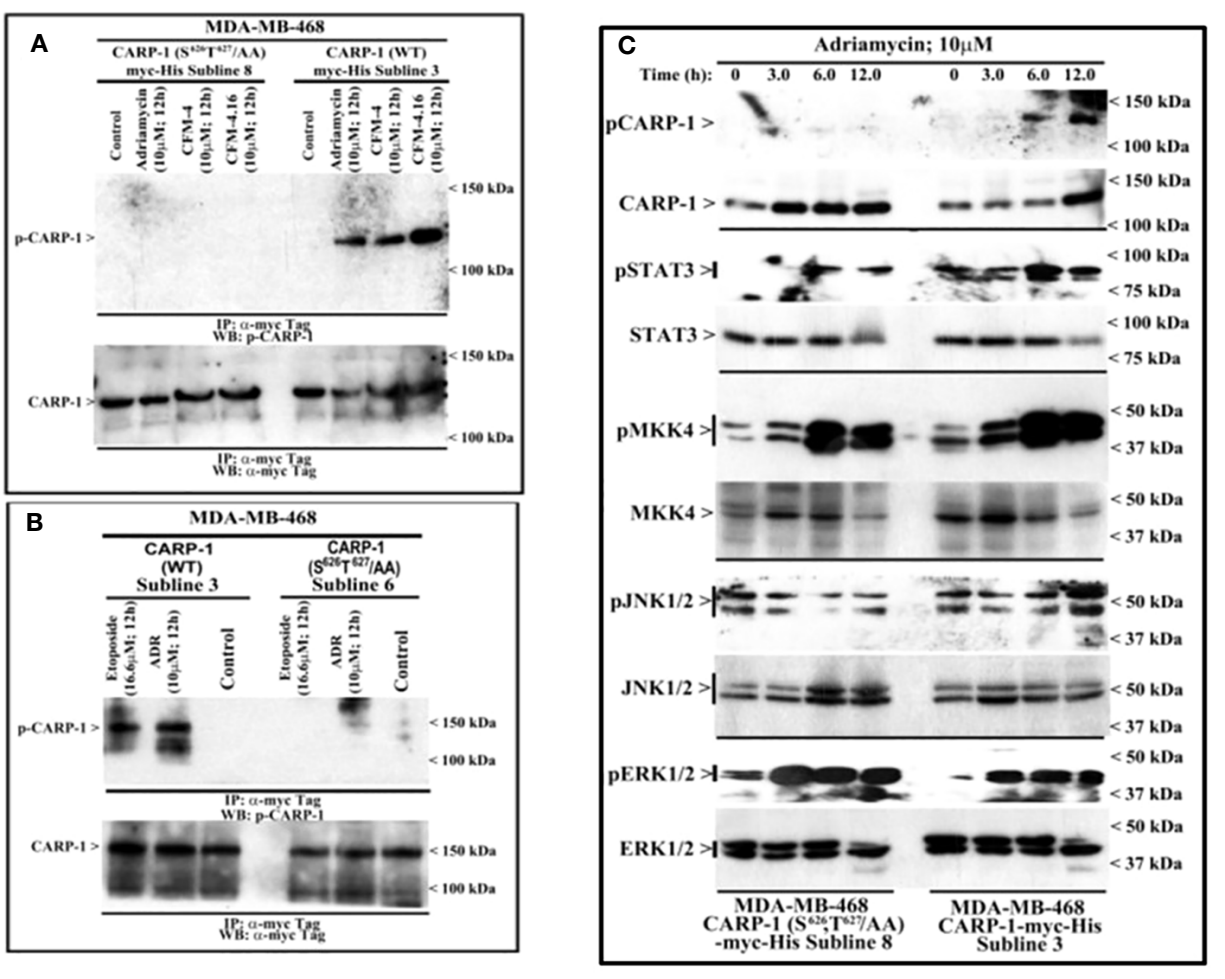
DNA damaging agents induce CARP-1 phosphorylation at T^627^ and activate various transducers of cell growth and survival signaling. **(A-C)**, indicated cell lines stably expressing myc-His-tagged wild-type CARP-1 or CARP-1 (S^626^T^627^/AA) mutant were treated with DMSO (Control), or with the noted dose and time of indicated agents. In **(A, B)**, Protein complexes were immunoprecipitated (I.P.) with anti-myc tag antibodies followed by the analysis of the immunocomplexes by western blot (W.B.) using anti-phospho-CARP-1 (Upper blot), and anti-myc tag (Lower blot) antibodies. In **(C)**, Cell lysates were analyzed by W.B. for levels of phosphorylated and total CARP-1, STAT3, MKK4, JNK1/2, and ERK1/2 as described in Methods. Arrowheads on the left or right side indicate presence of proteins or molecular weight markers, respectively.

To clarify the MAPK/SAPK that functions to phosphorylate CARP-1 in the presence of genotoxic stress, we first generated and characterized rabbit polyclonal antibodies utilizing T^627^-phosphorylated CARP-1 peptide as antigen as described in methods. The serum from rabbit with high titer was affinity purified, and utilized in subsequent studies. The WB analyses showed a robust CARP-1 T^627^ phosphorylation in cell lysates derived from MDA-MB-468 HBC cells expressing myc-His-CARP-1 (WT) that were treated with ADR, Etoposide, CFM-4, or CFM-4.16 compounds when compared with untreated control cells (**Figures 2A, B**). No CARP-1 phosphorylation was detectable in the protein lysates that were derived from MDA-MB-468 HBC cells expressing myc-His-CARP-1 (S^626^T^627^/AA) that were treated with either ADR, Etoposide, or CFM compounds (**Figures 2A, B**). Moreover, a time-dependent increase in phosphorylation of MKK4, JNK1/2, and ERK1/2 kinases as well as STAT3 protein occurred in ADR-treated MDA-MB-468 HBC cells expressing myc-His-CARP-1 (WT), while phosphorylation of JNK1/2 and STAT3, but not ERK1/2 or MKK4, was diminished in MDA-MB-468 HBC cells expressing myc-His-CARP-1 (S^626^T^627^/AA) that were treated with ADR (**Figure 2C**). These data suggest that ADR-induced cell growth and survival signaling by JNK1/2 and STAT3 likely involves CARP-1 phosphorylation.

### Various signaling transducers interact with epitope surrounding CARP-1 T^627^

Since our kinase assays (**Table 1**) indicated phosphorylation of CARP-1 (611–640) peptide by JNK1/2/3, ERK1/2, and p38γ, p38δ but not p38α/β kinases, we next determined the kinase(s) that target CARP-1 phosphorylation in the presence of ADR. We first conducted a high-throughput analyses of proteins interacting with CARP-1 epitope surrounding T^627^. We carried out Kinase- catalyzed Cross Linking and Streptavidin Purification (K-CLASP) (23–25) with an aim to identify the kinases and associated proteins that interact with CARP-1 T^627^ phosphosite. ADR-treated HeLa cell lysates were incubated with biotin-labelled CARP-1 (614–638) or CARP-1 (614-638; S^626^, T^627^/AA) and ATP-arylazide (ATP-ArN3) as a kinase co-substrate to crosslink the phosphopeptides with kinases and interacting proteins covalently with UV light as detailed in Methods. Peptide-bound complexes were then purified using streptavidin resin and interacting proteins were identified using liquid chromatography-tandem mass spectrometry (LC-MS/MS). We found 252 possible kinases and associated proteins that were enriched at least 1.2-fold in their interaction with CARP-1 (614–638) versus CARP-1 (614-638; S^626^,T^627^/AA) peptide in 3 out of 4 trials. A list of selected target proteins that interacted with CARP-1 (614–638) peptide is shown in **Table 3**. Of note is the fact that CCAR2 (DBC1; a CARP-1 paralog), Filamin C, and BRAF have been demonstrated to interact with CARP-1 in prior studies by us and others (7, 16, 26, 27). CARP-1 (614–638) peptide also interacted with MAPK12/p38γ suggesting that MAPK12/p38γ could be the kinase among the MAPKs noted in **Table 1** above that functions to transduce CARP-1 T^627^ phosphorylation by ADR.

**Table 3:** List of selected signal transducers that interact with CARP-1 (614–638) peptide that were identified through K-CLASP methodology as described in methods.

CARP-1 (614–638)-interacting Target Protein Name	Target Protein Abbreviation	# of Trials
S-phase kinase-associated protein 1 (Part of the SCF Ubiquitin Ligase)	SKP1	4
Filamin C	FLNC	4
Ribosomal protein S6 kinase alpha-4;Ribosomal protein S6 kinase alpha-5	RPS6KA4;RPS6KA5	4
5-AMP-activated protein kinase catalytic subunit alpha-1	PRKAA1	4
N-terminal kinase-like protein	SCYL1	3
Mitogen-activated protein kinase 12/p38γ	MAPK12/p38γ	3
Protein-tyrosine kinase 2-beta	PTK2B	3
Extracellular serine/threonine protein kinase FAM20C	FAM20C	3
RAF proto-oncogene serine/threonine-protein kinase	RAF1 (c-Raf)	3
cAMP-dependent protein kinase type II-beta regulatory subunit;cAMP-dependent protein kinase type II-alpha regulatory subunit	PRKAR2B;PRKAR2A	3
Bifunctional ATP-dependent dihydroxyacetone kinase/FAD-AMP lyase (cyclizing); ATP-dependent dihydroxyacetone kinase;FAD-AMP lyase (cyclizing)	DAK	3
Serine/threonine-protein kinase WNK3	WNK3	3
Signal transducer and activator of transcription 3	STAT3	3
Histone deacetylase 1	HDAC1	3
Histone deacetylase 7	HDAC7	3
E3 ubiquitin-protein ligase UBR4	UBR4	3
Cell cycle and apoptosis regulator protein 2	CCAR2	3
Serine/threonine-protein kinases (STK) 26; 25; 24 (36kD subunit); 24 (12 kD subunit)	STK26;STK25;STK24	2
Rho-associated protein kinase 2	ROCK2	2
Cyclin-dependent kinase 4;Cyclin-dependent kinase 6	CDK4; CDK6	2
Nucleoside diphosphate kinase A;Nucleoside diphosphate kinase B	NME1;NME2	2
Myosin light chain kinase, smooth muscle;Myosin light chain kinase, smooth muscle, deglutamylated form	MYLK	2
Alpha-protein kinase 3	ALPK3	2
A-kinase anchor protein 9	AKAP9	2
Nuclear receptor corepressor 1	NCOR1	2
Proliferating cell nuclear antigen	PCNA	2
Serine/threonine-protein kinase 38-like	STK38L	2

Proteins highlighted in yellow have been demonstrated previously by us and others or in current studies to interact with CARP-1.

**Figure 3:**
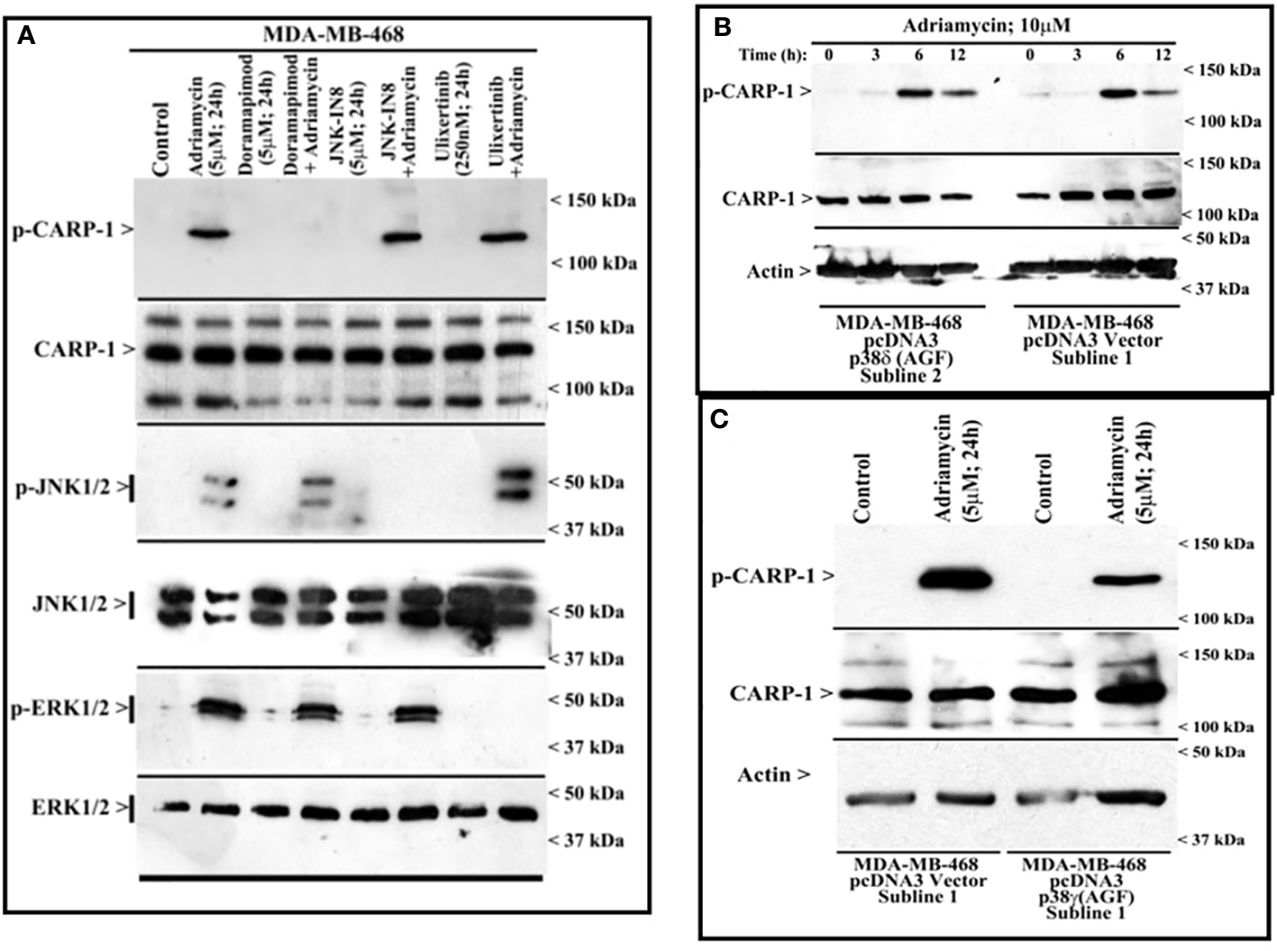
MAPK/SAPK p38γ regulates Adriamycin-induced CARP-1 phosphorylation. Wild-type HBC cells **(A)** and HBC cells stably expressing vector, catalytically inactive mutant of p38δ **(B)**, or catalytically inactive mutant of p38γ **(C)** were treated indicated agent for noted time and dose. Cell lysates were then analyzed by western blot (W.B.) for levels of phosphorylated and total CARP-1, JNK1/2, ERK1/2 proteins as described in Methods. The W.B membranes in panels B and C were probed with anti-actin antibodies to assess protein loading. Arrowheads on the left or right side of each blot in panels **(A-C)** indicate presence of proteins or molecular weight markers, respectively.

We then validated binding of various signal transducers that interacted with and/or phosphorylated CARP-1 T^627^. We conducted co-immunoprecipitation-Western blot analyses utilizing lysates from HBC cells that stably expressed myc-His-tagged CARP-1, CARP-1 (S^626^T^627^/AA), or CARP-1 (Δ600-650) proteins as detailed in Methods. As shown in **Supplementary Figures S6A-C**, CARP-1 interacted with JNK1/2, p38γ, p38δ, ERK1/2, MEK1/2, H2AX, and STAT3 proteins. Moreover, as summarized in **Table 4**, CARP-1 (WT) and CARP-1 (S^626^T^627^/AA) but not CARP-1 (Δ600-650) interacted with JNK1/2, H2AX, and Filamin C. However, only CARP-1 (WT) protein interacted with ERK1/2, RIPK1, CDK4/6, MEK1/2, STAT3, p38γ, and p38δ. Neither CARP-1 (WT) nor its mutant proteins interacted with MKK4, MSK2, and p38α/β (**Table 4**). As also expected, ADR treatment caused a robust phosphorylation of CARP-1 but not its S^626^T^627^/AA mutant protein (**Supplementary Figure S6D**). Of note is that CARP-1 interactions with STAT3 and ERK1/2 were appreciably diminished in ADR-treated cells when compared with their interactions in the untreated control cells (**Supplementary Figure S6D**). Since ADR-induced CARP-1 phosphorylation at T^627^ while substitution of CARP-1 S^626^T^627^ to alanines interferes with CARP-1 interactions with STAT3, RIPK1, and ERK1/2 (**Supplementary Figures S6D, E**), it remains to be determined whether phosphorylation of CARP-1 T^627^ or substitution of CARP-1 T^627^ to alanine induces a conformational change that results in diminished interaction of CARP-1 with STAT3, RIPK1, and ERK1/2 proteins. Moreover, given that ADR-treated cells expressing CARP-1 (S^626^T^627^/AA) or CARP-1 (S^626^T^627^T^629^/AAA) have increased viabilities when compared with their counterparts expressing wild-type CARP-1 (**Figure 1D**, **Supplementary Figures S1B, D**), and ADR treatment also caused robust phosphorylation of MAPKs ERK1/2 (**Figure 3A**), it is possible that ADR-induced cell growth and survival signaling by MAPK pathway is regulated in part by P38γ-mediated CARP-1 phosphorylation. Whether and to the extent, ADR-induced, p38γ-mediated CARP-1 phosphorylation is also involved in activation of CDK4/6 to regulate cell proliferation remain to be clarified.

**Table 4:** List of CARP-1-interacting signal transducers identified by coimmunoprecipitation western blot (co-IP-W.B.) experiments utilizing protein lysates derived from cells stably expressing CARP-1-myc-His, CARP-1 (S^626^T^627^/AA)-myc-His, or CARP-1 (Δ600-650)-myc-His proteins.

Target Kinase/ Protein	CARP-1 (WT) Binding	CARP-1 S^626^T^627^/AA Binding	CARP-1 (Δ600-650) Binding
cRAF/b-RAF	Yes	Yes	Yes
JNK1/2	Yes	Yes	No
H2AX	Yes	Yes	No
FLNC	Yes	Yes	No
ERK1/2	Yes	No	No
RIPK1	Yes	No	No
CDK4/6	Yes	No	No
MEK1/2	Yes	No	No
STAT3	Yes	No	No
p38γ	Yes	No	No
p38δ	Yes	No	No
MKK4	No	No	No
MSK2	No	No	No
P38α/β	No	No	No

### MAPK/SAPK p38γ phosphorylates CARP-1 T^627^ in the presence of DNA damaging agents

**Figure 4:**
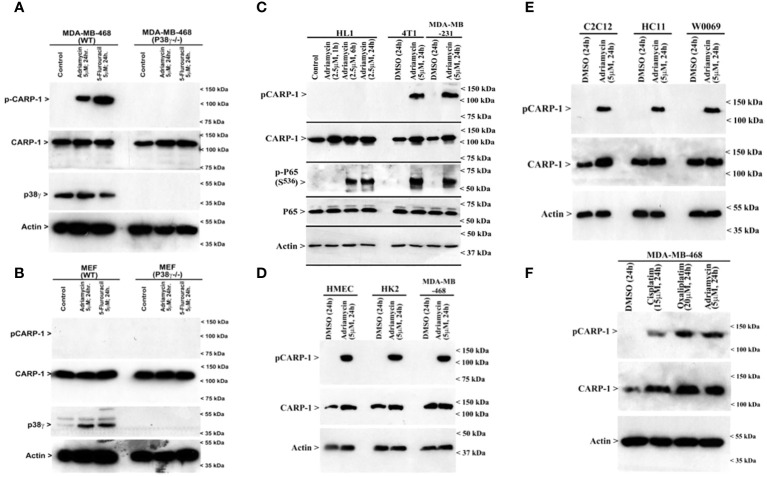
p38γ is required for genotoxic chemotherapy-induced CARP-1 phosphorylation. **(A-F)**, indicated cell lines were treated with DMSO (Control), or with the noted dose and time of indicated agents. Cell lysates were analyzed by western blot (W.B.) using antibodies for phospho-CARP-1, total CARP-1, p38γ, phospho-p65, total p65, and actin as described in Methods. Arrowheads on the left or right side of each panel indicate presence of proteins or molecular weight markers, respectively. Cell lines utilized are detailed in **Supplementary Table S3**. HK2, renal epithelial cells; HMECs, Human Mammary Epithelial Cells; MEFs, Mouse Embryonic Fibroblasts; HC11, murine breast epithelial cells; C2C12, murine myoblast cells; HL1, murine cardiomyocytes; 4T1, murine breast cancer cells; W0069, murine BRCA-deficient mammary tumor cells.

Next, we treated wild-type MDA-MB-468 HBC cells with ADR, pan-p38 inhibitor Doramapimod (28), JNK inhibitor JNK-1N8 (11, 29), or ERK inhibitor Ulixertinib (16, 30) as single agents or ADR in combination with respective MAPK/SAPK inhibitor as detailed in Methods. The cell lysates were analyzed by WB for presence of T^627^ phosphorylated CARP-1. This experiment revealed that Doramapimod, but not JNK-IN8 or Ulixertinib, abrogated ADR-induced CARP-1 phosphorylation (**Figure 3A**). Since p38α and β isoforms failed to phosphorylate CARP-1 (611–640) peptide in kinase assays, we then determined whether ADR-dependent CARP-1 phosphorylation involved p38γ and/or p38δ isoforms. We utilized MDA-MB-468 HBC cells that have stable expression of vector, catalytically inactive p38γ (p38γ AGF), or catalytically inactive p38δ (p38δ AGF) that are described above (**Supplementary Figures S3F, K**). Although ADR provoked a robust increase in CARP-1 phosphorylation in vector-expressing HBC cells, ADR-dependent CARP-1 T^627^ phosphorylation was diminished in HBC cells expressing catalytically inactive p38γ but not in HBC cells expressing catalytically inactive p38δ (**Figures 3B, C**). These data suggest that p38γ transduces ADR-dependent CARP-1 phosphorylation, and would be consistent with a robust phosphorylation of CARP-1 (611–640) peptide by p38γ that was noted in the kinase assays summarized in **Table 1**. We next investigated whether p38γ was required for genotoxic chemotherapy-induced CARP-1 phosphorylation. For this purpose, we utilized p38γ-null mouse embryonic fibroblasts (MEFs, 31) that were generously provided by Dr. Ana Cuenda, (Department of Immunology and Oncology, Centro Nacional de Biotecnología (CSIC), Madrid, Spain). In addition, we generated and characterized p38γ-null MDA-MB-468 HBC cells as described in methods. As expected, ADR induced CARP-1 phosphorylation in MDA-MB-468, MDA-MB-231, and 4T1 Triple-negative breast cancer cells (**Figures 4A, C**). Absence of p38γ in MDA-MB-468 HBC cells abrogated genotoxic therapy-induced CARP-1 phosphorylation, demonstrating p38γ requirement for CARP-1 T^627^ phosphorylation by genotoxic chemotherapy. Moreover, genotoxic chemotherapies induced CARP-1 phosphorylation in additional HBC cells, human mammary epithelial cells (HMECs), human renal epithelial HK2 cells (13), as well as murine C2C12 myoblast cells (32), HC11 murine breast epithelial cells (33), and W0069 BRCA-deficient mouse mammary tumor cells (34) but failed to promote CARP-1 phosphorylation in wild-type murine MEFs as well as murine cardiomyocytes (**Figures 4B, C**). The reason(s) for this intriguing absence of ADR-induced CARP-1 phosphorylation in murine MEFs and cardiomyocytes remain to be clarified. Nevertheless, our data in **Figures 1**-**4** collectively suggest that SAPK p38γ-mediated CARP-1 T^627^ phosphorylation functions to transduce apoptosis signaling by genotoxic chemotherapy.

#### Loss of p38γ abrogates genotoxic chemotherapy induced cell growth inhibition

**Figure 5:**
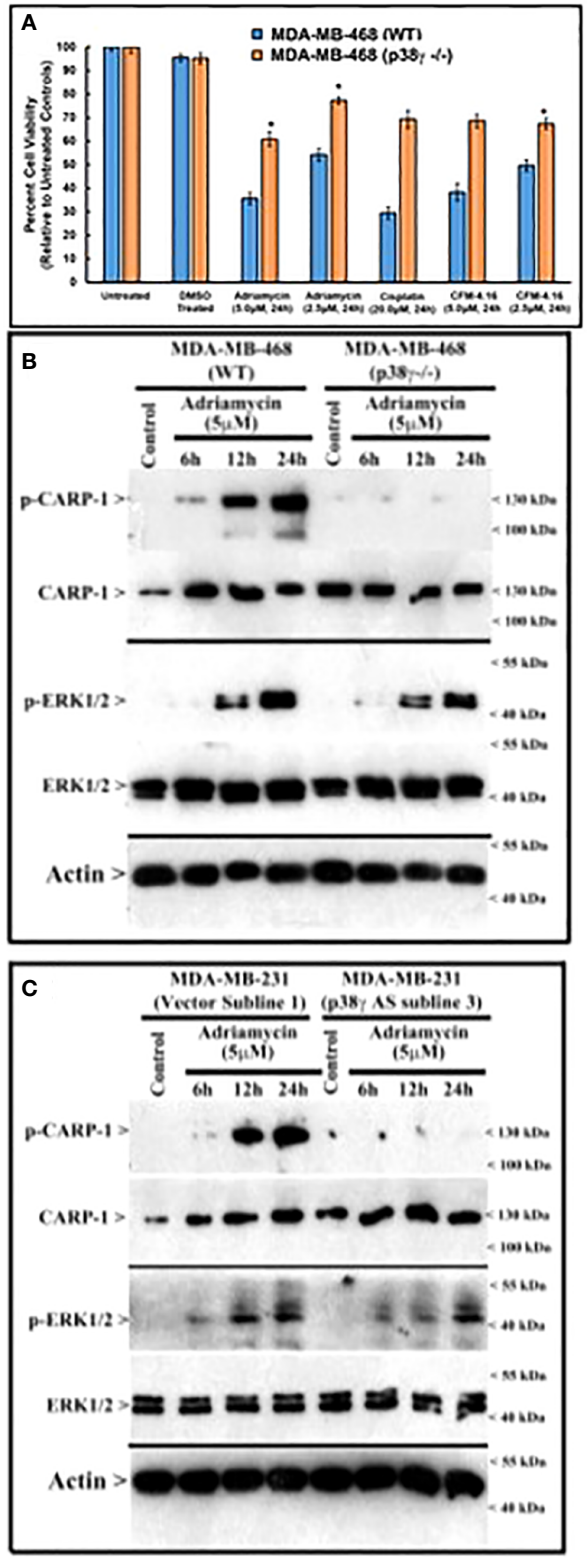
MAPK/SAPK p38γ promotes CARP-1 phosphorylation in Adriamycin-treated HBC cells. **(A)**, Cell viability was determined by MTT assay following treatments of cells with vehicle/DMSO (Control) or indicated times and dose of various agents. The columns in histogram indicate percent of live/viable cells relative to their DMSO-treated controls and represent means of three independent experiments; bars, S.E. **(B, C)**, indicated HBC cells (WT, p38γ -/-, and cell lines stably expressing vector or p38γ antisense; noted as AS) were treated with DMSO (Control), or with the noted dose and times of Adriamycin. Cell lysates were analyzed by western blot (W.B.) for levels of phosphorylated and total CARP-1, phospho-CARP-1, phospho-ERK1/2, ERK1/2, and actin proteins as described in methods. Arrowheads on the left or right side indicate presence of proteins or molecular weight markers, respectively.

Since loss of p38γ abrogated CARP-1 T^627^ phosphorylation by genotoxic chemotherapy (**Figure 4A**), we next investigated whether absence of p38γ would confer resistance to genotoxic chemotherapy. For this purpose, we utilized p38γ-/- MDA-MB-468 cells (**Figure 4A**). Parental and p38γ-/- cells were treated with Adriamycin, Cisplatin, Gemcitabine, and experimental compound CFM-4.16. Loss of p38γ protected cells from growth inhibition by various DNA damage-inducing agents (**Figure 5A**; **Supplementary Figure S7D**). Next, western blot analyses were carried out to determine expression of phosphorylated CARP-1 and ERK proteins following treatments of MDA-MB-468 (WT), p38γ-/-, and p38γ AS (p38γ knockdown) cells with various DNA damage-inducing agents including Adriamycin, Cisplatin, 5-fluorouracil, CFM-4.16, and Gemcitabine as noted in methods. All of these agents induced phosphorylation of CARP-1 in MDA-MB-468 (WT) cells but not in p38γ-/- and p38γ AS cells (**Figures 5B, C**; **Supplementary Figures S7A-C, E**). ERK1/2 activation was moderately diminished in p38γ-/- and p38γ AS cells when compared with their WT counterparts in the presence of all the agents except Cisplatin at the 6h treatments periods. Although CFM-4.16 treatments caused elevated activation of ERK1/2 in p38γ-/- cells relative to its activation in CFM-4.16-treated WT cells, ERK1/2 phosphorylation was diminished in p38γ-/- and p38γ AS when compared with their WT counterparts that were treated with Adriamycin and Cisplatin but not 5-fluorouracil and Gemcitabine. Interestingly, as shown in **Supplementary Figure S8A**, loss of p38γ did not protect cells from inhibitory effects of Paclitaxel (a chemotherapy that does not induce DNA damage). Moreover, neither serum deprivation nor treatments with Paclitaxel induced CARP-1 phosphorylation in MDA-MB-468 (WT) and in MDA-MB-468 p38γ-/- cells, respectively (**Supplementary Figures S8B**, **S9**). Paclitaxel treatments resulted in a moderate decrease in levels of ERK1/2 phosphorylation in MDA-MB-468 p38γ-/- cells versus MDA-MB-468 (WT) cells (**Supplementary Figure S8B**). These data collectively suggest that p38γ is required for DNA damage-induced T^627^ phosphorylation of CARP-1 and elevated levels of CARP-1 phosphorylation transduce inhibitory effects of DNA damage-inducing chemotherapy. Paclitaxel, a chemotherapy that functions by stabilizing microtubules without inducing DNA damage, inhibits cell growth but does not induce CARP-1 phosphorylation. As serum deprivation and Paclitaxel also caused elevated CARP-1 levels in WT MDA-MB-468 cells, it is likely that absence of T^627^ phosphorylated CARP-1 also functions to support cell growth inhibition in signaling contexts different from that involving CARP-1 T^627^ phosphorylation. Together with our prior report demonstrating involvement of CARP-1 interaction with NF-κB kinase subunit IKKγ/NEMO in promoting DNA damage-induced p65 activation (8), our current studies reveal a biphasic signaling by CARP-1 where CARP-1 signals in cell growth and survival in part by activating NF-κB signaling while the growth inhibitory signaling by CARP-1 is dependent in part on its p38γ-mediated T^627^ phosphorylation.

#### CARP-1 phosphorylation associates with tumors from patients treated with radiation or endocrine therapies

**Figure 6:**
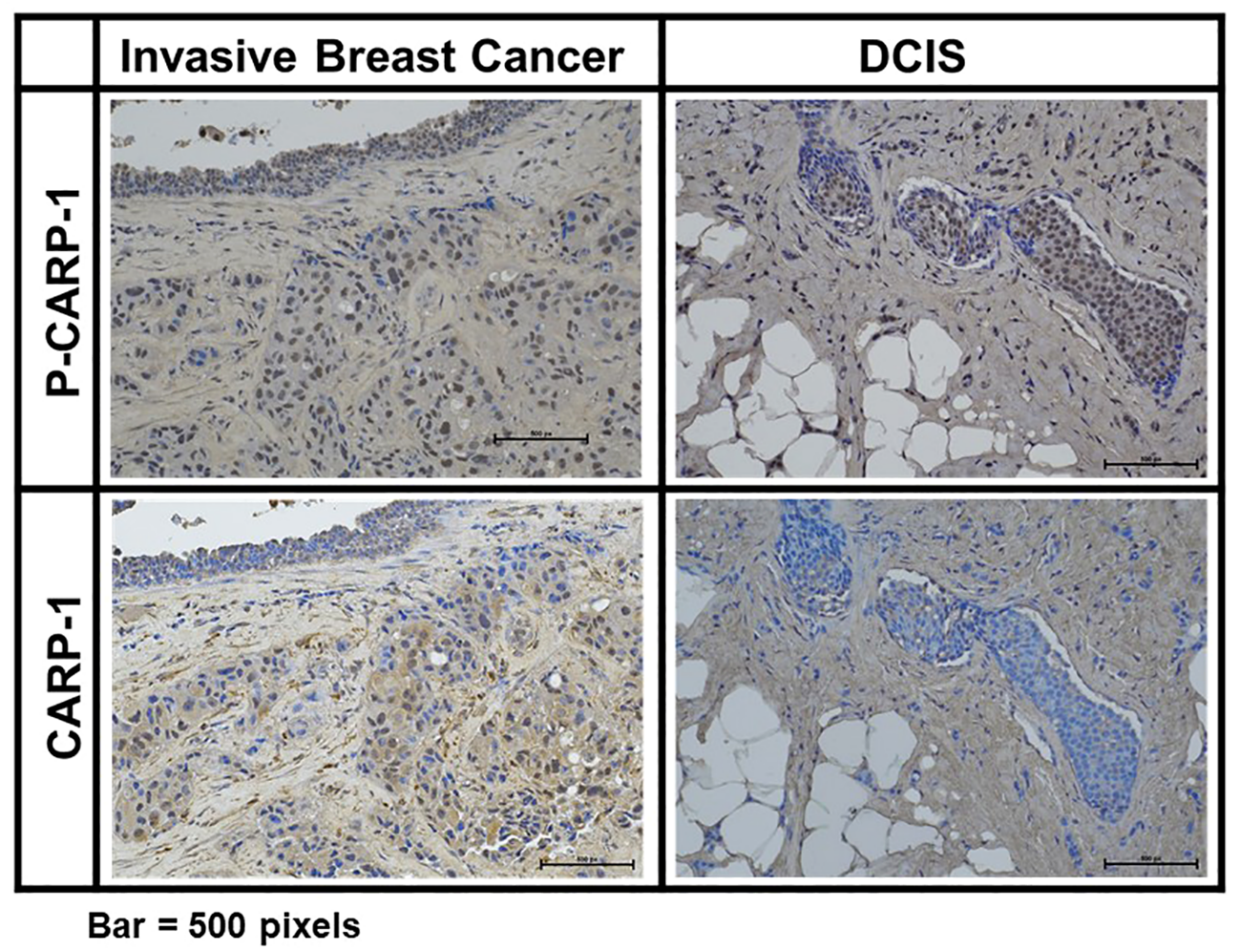
Expression of CARP-1 in breast ductal carcinoma in situ. Immuno-histochemical staining for presence of phospho-CARP-1 (P-CARP-1) or CARP-1 proteins in the DCIS tumors was carried out as detailed in Methods. Bar, 500 pixels.

Prior reports have indicated induction of p38γ expression by Ras oncogene that functions to promote Ras-dependent transformation and invasion by cancer cells (35). P38γ was required for malignant growth and its expression was elevated in primary tumor tissues (35–37). P38γ promoted epithelial-to-mesenchymal transition (EMT), and p38γ-dependent phosphorylation of topoisomerase II resulted in increased sensitivity of breast cancer cells to DNA damage-inducing therapies that target topoisomerase II (38, 39). On the other hand, p38γ signaling also contributed to resistance to therapies that target estrogen receptor (ER) and PARP proteins (40, 41), while p38γ was required for transduction of γ-radiation induced stress response (42, 43). Recent reports revealed P38 MAPK was expressed and activated (phosphorylated) at a higher level in ER-positive primary breast tumors when compared with ER-negative counterparts (44, 45). In light of our findings demonstrating CARP-1 phosphorylation by p38γ, and the fact that p38γ is overexpressed in primary colon cancers, and is a potential therapeutic target for triple-negative breast cancer (TNBC), we next investigated whether CARP-1 phosphorylation was altered in primary breast cancers. Our immunohistochemical analyses revealed CARP-1 phosphorylation in two representative specimens of invasive ductal carcinoma but not in normal ducts (**Figure 6**). On the basis of this data, we next conducted immunohistochemical analyses for phospho-CARP-1 expression in a breast cancer tumor microarray containing 504 primary breast cancers as detailed in Methods. Although all the tumors stained for presence of CARP-1, a subset of 83 tumors stained for phospho-CARP-1 (**Supplementary Table S8**). Interestingly, phospho-CARP-1 presence correlated with patients who received radiation (p= <0.041) or endocrine therapies (p= <0.038). Since radiation and anti-estrogen signaling activate p38γ (41, 42) our proof-of-concept findings from the TMA analysis suggest that p38γ-dependent CARP-1 T^627^ phosphorylation could be a potential predictor of responses to radiation or hormonal therapies in a subset of breast cancers.

## Discussion

In this report we describe for the first time that CARP-1/CCAR1 functions as a regulator of genotoxic chemotherapy induced cell growth inhibitory signaling that involves T^627^ phosphorylation of CARP-1 by p38γ MAPK/SAPK (**Figures 3**, **5**). Our findings would suggest genotoxic stress-induced, p38γ-mediated T^627^ phosphorylation of CARP-1 likely contributed to apoptosis as cells expressing CARP-1 (S^626^, T^627^, T^629^/AAA) mutant protein had diminished activation of various caspases when treated with genotoxic chemotherapy (**Figure 1E**).

One of the key and significant finding of our current study is that CARP-1 T^627^ is a novel substrate for phosphorylation by p38γ during genotoxic chemotherapy-induced apoptosis signaling. P38γ belongs to a family of serine/threonine kinases that function to transduce mitogenic as well as stress signaling, and are categorized as MAPKs/SAPKs. A number of prior reports have indicated p38γ as a potential oncogene in models of triple-negative breast, pancreatic, liver, and colon cancers (36–41, 43–46). Although p38γ functions to regulate stem cell expansion, metastasis, and AP-1 signaling pathways, a recent three-dimensional (3D) search revealed a higher degree of structural similarity among p38γ and active CDK1 and CDK2 kinases, as both the CDK1 and p38γ induce phosphorylation at the same residues in the Rb protein (47). Interestingly, our data in **Supplementary Figure S3** and **Supplementary Table S7** indicate that like Rb protein, CARP-1 T^627^ could also be substrate for the p38γ and CDK1 kinases. As CARP-1 is also a co-activator of cell-cycle regulatory APC/C E3 ligase (7), it remains to be clarified whether p38γ phosphorylation of CARP-1 T^627^ or a different, yet to be characterized S/T residue(s) functions to promote APC/C E3 ligase activation for cell cycle progression, and together with p38γ-dependent Rb phosphorylation, p38γ kinase contributes to cell cycle progression, cell proliferation, growth, and oncogenesis. However, MKK6-p38γ cascade was also involved in gamma-radiation-induced cell cycle arrest (42), and the fact that substitution of CARP-1 T^627^/A abrogates cell growth inhibition by genotoxic stress (**Figure 1**) further support our hypothesis that p38γ transduces cell cycle inhibitory and apoptotic signaling following genotoxic stress in part by phosphorylating CARP-1 T^627^.

Our TMA analyses intriguingly revealed CARP-1 T^627^ phosphorylation in breast cancer biopsies from patients who received radiation or endocrine therapies. These findings would suggest for additional signaling contexts for CARP-1 T^627^ phosphorylation. Given that MAPK/SAPKs JNK1/2 and p38δ also phosphorylated at CARP-1 T^627^ site as indicated by our kinase profiling analyses (**Table 1**), it remains to be determined whether and to the extent endocrine therapy-induced CARP-1 T^627^ phosphorylation also involves p38γ or a different MAPK/SAPK. On the other hand, genotoxic chemotherapy failed to induce CARP-1 T^627^ phosphorylation in murine embryonic fibroblasts (MEFs) and cardiomyocytes albeit p38γ was upregulated in genotoxic chemotherapy-exposed MEFs (**Figure 4**). Whether genotoxic chemotherapy-induced cell growth inhibitory effects in murine embryonic fibroblasts (MEFs) and cardiomyocytes involve phosphorylation of different S/T residue(s) of CARP-1 that are targeted by p38γ or involve a different S/T phosphorylation targeted by another MAPK/SAPK(s) is also unclear. Overall, CARP-1 is a novel substrate of p38γ and possibly other MAPK/SAPK(s) for its T^627^ phosphorylation and subsequent transduction of signaling induced by genotoxic stress and other signaling contexts.

Genotoxic stress-induced ATM kinase activates the NF-κB pathway, which regulates genes involved in inhibition of apoptosis and promotion of cell survival. ATM-induced NF-kB signaling not only permits normal cells to repair DNA but also enables malignant cells to resist genotoxic therapy. Our previous report revealed CARP-1 binding with IKKγ (aka NEMO) regulated genotoxic chemotherapy-induced activation of canonical NF-κB and secretion of pro-inflammatory cytokines (8). CARP-1 binding with NEMO facilitated ATM kinase-mediated NEMO phosphorylation in the nucleus and consequent activation of cytosolic IKK complex followed by activation and nuclear import of p65/RelA. Disruption of CARP-1binding with NEMO resulted in attenuation of p65 phosphorylation and inhibition of NF-κB signaling. We have previously noted CARP-1 interaction with RIPK1 (15). Here we find that CARP-1 interacts with RIPK1 in cells expressing wild-type CARP-1 but this interaction is abrogated in cells expressing CARP-1 (S^626^T^627^/AA) mutant (**Supplementary Figure S6E**). Moreover, CARP-1 interaction with RIPK1 is diminished in ADR-treated cells expressing wild-type CARP1 relative to that noted in their untreated counterparts (**Supplementary Figure S6E**).

RIPK1 is implicated in controlling NF-κB as well as cell death in response to DNA damage. Extensive DNA lesions promote p53-independent two sequential NF-κB activation phases. In the first phase ATM and NEMO promote TNFα production via IKK/RelA signaling. The second phase involves TNFα/TNFR signaling to drive RIPK1 phosphorylation followed by JNK3/MAPK10-IL-8/FADD-mediated caspase-8 activation (48). We previously noted that phosphorylation of NEMO and p65/RelA but not JNK1/2 occurs when cells are exposed to genotoxic insult for 1h (8) while T^627^ phosphorylation of CARP-1 and JNK1/2 activation occurs when cells are exposed to Adriamycin for 6h or longer (**Figure 2C**). Thus, in the context of early DNA damage, ATM likely activates NF-κB signaling through a dynamic CARP-1/NEMO/RIPK1/IKK/p65 cascade to promote survival and DNA repair. DNA damage over extended period likely results in above ATM-regulated apoptotic signaling through TNFα/JNK1/2/caspase-8 cascade as well as p38γ-mediated phosphorylation of CARP-1 (**Figure 3**). Loss of p38γ abrogates inhibitory effects of genotoxic chemotherapy. Although CARP-1 also interacts with caspase-8 and FADD (15) in untreated as well as CFM-4 (parent compound of CFM-4.16 utilized in this report), it appears likely that CARP-1 is a component of a dynamic, pro-apoptotic JNK/MAPK10-IL-8/FADD/caspase-8 proteome. Whether CARP-1 T^627^ phosphorylation contributes to apoptosis signaling by activating TNFα/JNK/RIPK1/Caspase-8 complex or interfering with TNFα/RIPK1/NEMO/p65 pathway remains to be clarified. However, the precise kinetics of genotoxic stress-induced, CARP-1-dependent cell growth, survival, and apoptosis signaling involving RIPK1 remain to be elucidated, and is a subject of our on-going studies.

In summary, we demonstrate that CARP-1 is a novel, endogenous regulator of chemotherapy-induced MAPK/SAPK p38γ that in turn phosphorylates CARP-1 T^627^ to promote apoptosis. These findings are consistent with previously noted biphasic roles of CARP-1 in regulating cell survival and chemotherapy resistance as well as cell growth inhibitory apoptotic signaling (1–4, 7–16). Our findings also reveal a novel biomarker potential of T^627^ phosphorylation of CARP-1 for radiation or endocrine therapy-treated breast cancers.

## Data availability statement

The original contributions presented in the study are included in the article/**Supplementary Material**. Further inquiries can be directed to the corresponding author.

## Ethics statement

Ethical approval was not required for the studies involving humans because Breast cancer tumor microarrays were utilized. The patient identifiers were not known. The studies were conducted in accordance with the local legislation and institutional requirements. The human samples used in this study were acquired from gifted from another research group. Written informed consent to participate in this study was not required from the participants or the participants’ legal guardians/next of kin in accordance with the national legislation and the institutional requirements. Ethical approval was not required for the studies on animals in accordance with the local legislation and institutional requirements because only commercially available established cell lines were used.

## Author contributions

JV: Data curation, Formal analysis, Investigation, Methodology, Writing – original draft, Software. MM: Formal analysis, Investigation, Methodology, Validation, Writing – original draft. IS: Writing – original draft. VC: Investigation, Writing – original draft, Methodology, Validation. SS: Formal analysis, Investigation, Writing – original draft. NA: Data curation, Formal analysis, Writing – original draft, Investigation, Software. EL: Writing – original draft, Data curation, Formal analysis, Methodology, Validation. HA: Writing – original draft, Investigation, Resources. MP: Conceptualization, Supervision, Writing – original draft, Project administration, Funding acquisition. AR: Conceptualization, Funding acquisition, Resources, Supervision, Writing – original draft, Writing – review & editing.
