# Supplementary material for: Phosphorylation of cell cycle and apoptosis regulatory protein-1 by stress activated protein kinase P38γ is a novel mechanism of apoptosis signaling by genotoxic chemotherapy
Source: Front Oncol. 2024 May 2;14:1376666. doi: 10.3389/fonc.2024.1376666 (PMC11096501; doi:10.3389/fonc.2024.1376666)
Supplement: Supplementary file 1 [file Image_1.pdf]

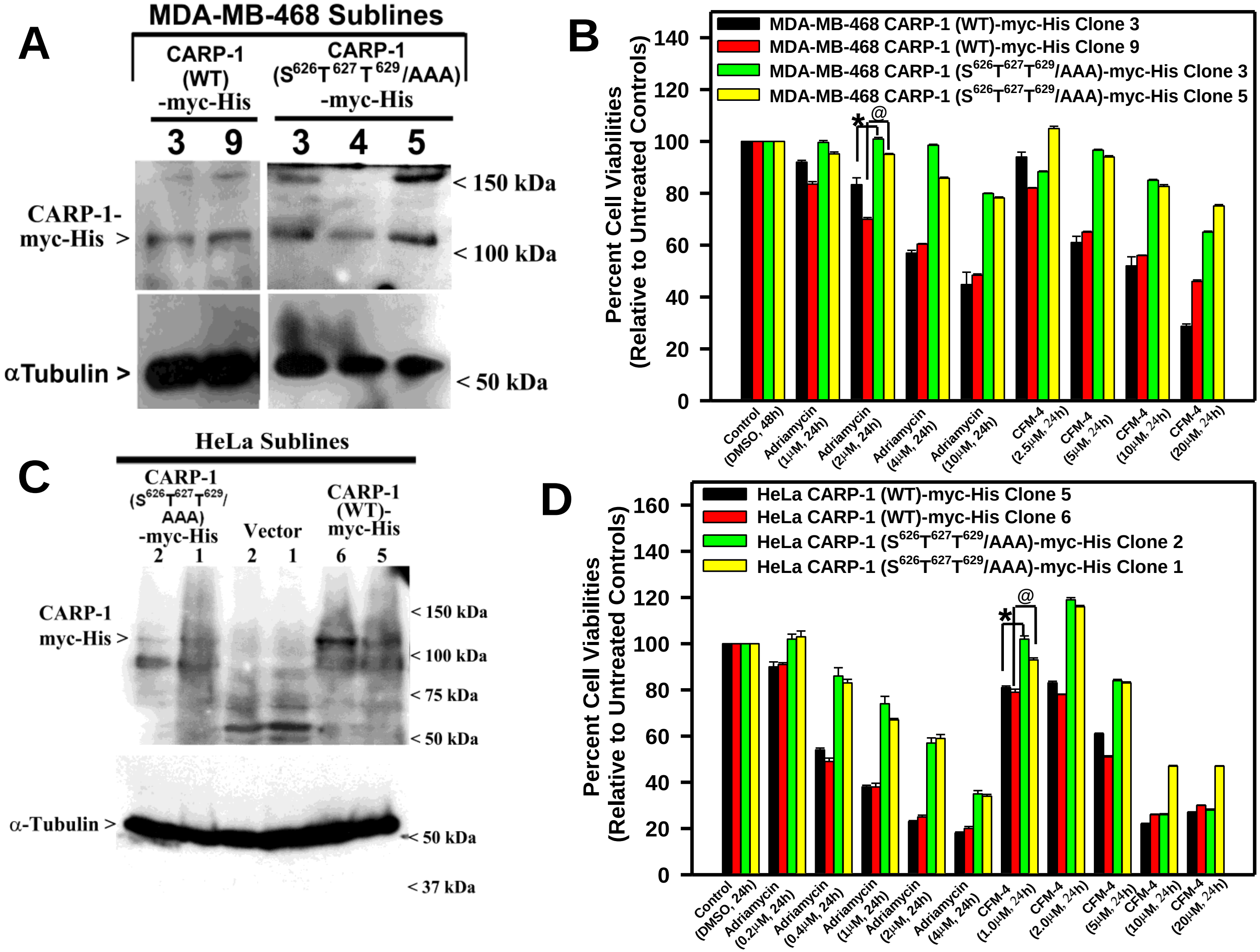

Supplementary Fig. 1: Substitution of CARP-1 Serine^{626}, Threonine^{627} and Threonine^{629} to alanines abrogates cell viability loss induced by DNA damaging agents. *A, C*. Noted cells were transfected separately with vector plasmid or indicated plasmids expressing CARP-1 (WT), CARP-1 ($S^{626}T^{627}T^{629}$/AAA) and neomycin-resistant, respective stable sublines were generated and characterized as described before (7, 8, 11). Expression of respective, transfected proteins was analyzed by W.B. (upper blots), and each membrane was probed with anti-α-Tubulin antibodies to assess protein loading (Lower blots). Arrowheads on the left or right side of each blot in panels indicate presence of proteins or molecular weight markers, respectively. *B, D*, Cell viability was determined by MTT assay following treatments of cells with vehicle/DMSO (Control) or indicated time and doses of various agents. The columns in each histogram indicate percent of live/viable cells relative to their DMSO-treated controls and represent means of three independent experiments. bars, S.E. B, D, *, @, - p <0.001 versus the corresponding vector cells as analyzed through students-t test. F, *-p<0.001 versus the corresponding CARP-1 (WT) expressing cells.

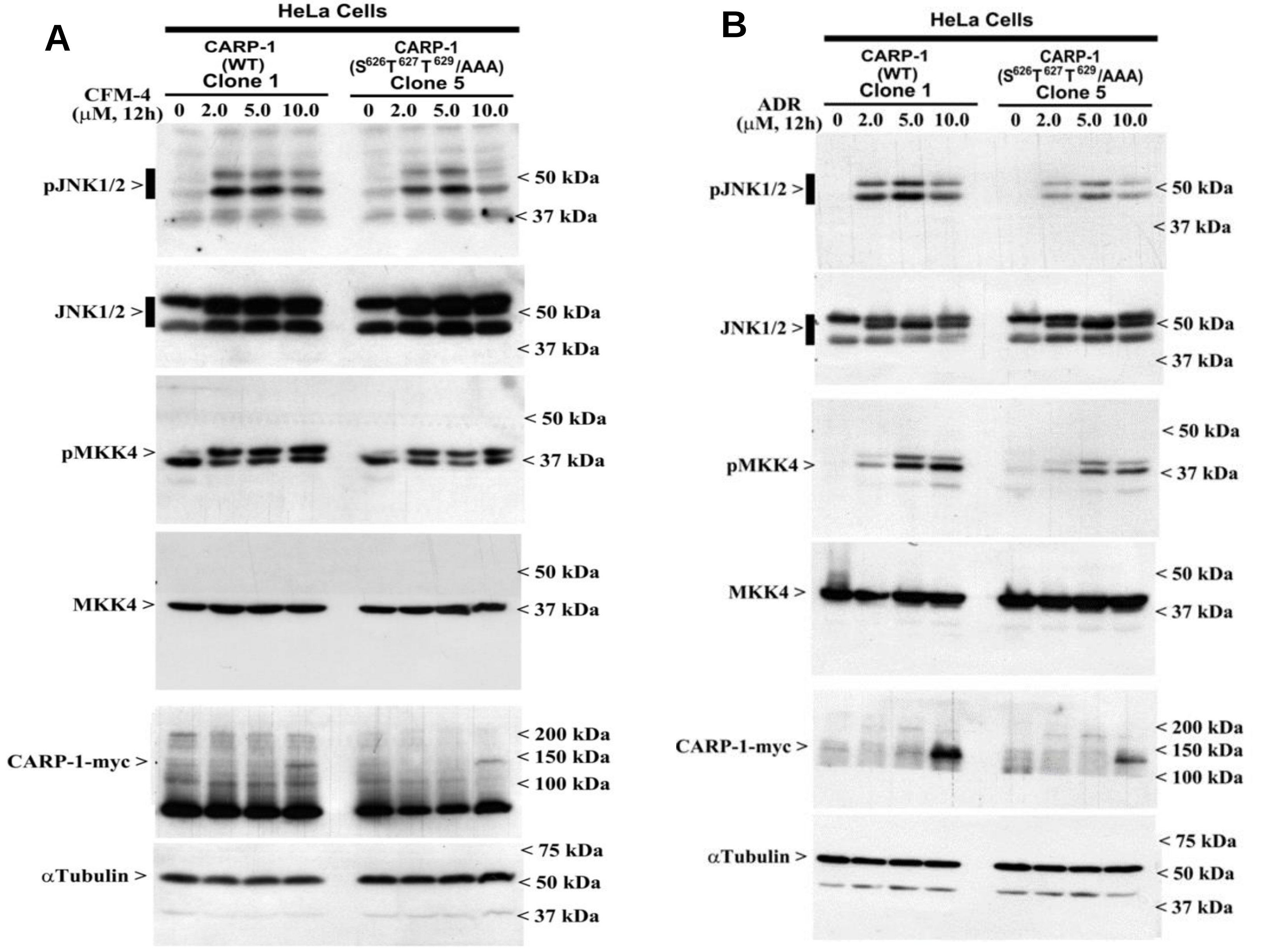

Supplementary Fig. 2: CFM-4 *(A) or* Adriamycin *(B)* activate MAPKs/SAPKs in cells expressing wild-type or mutant CARP-1. Indicated cells either untreated (0) or treated with noted doses and time of respective agents. W.B. analysis of the cell lysates was carried out using anti-CARP-1 (α2), anti-phospho-MKK4, anti-phospho JNK1/2, anti-MKK4, anti-JNK1/2, and anti-αtubulin antibodies. Arrowheads on the left or right side of each blot in each panel indicate presence of proteins or molecular weight markers, respectively.

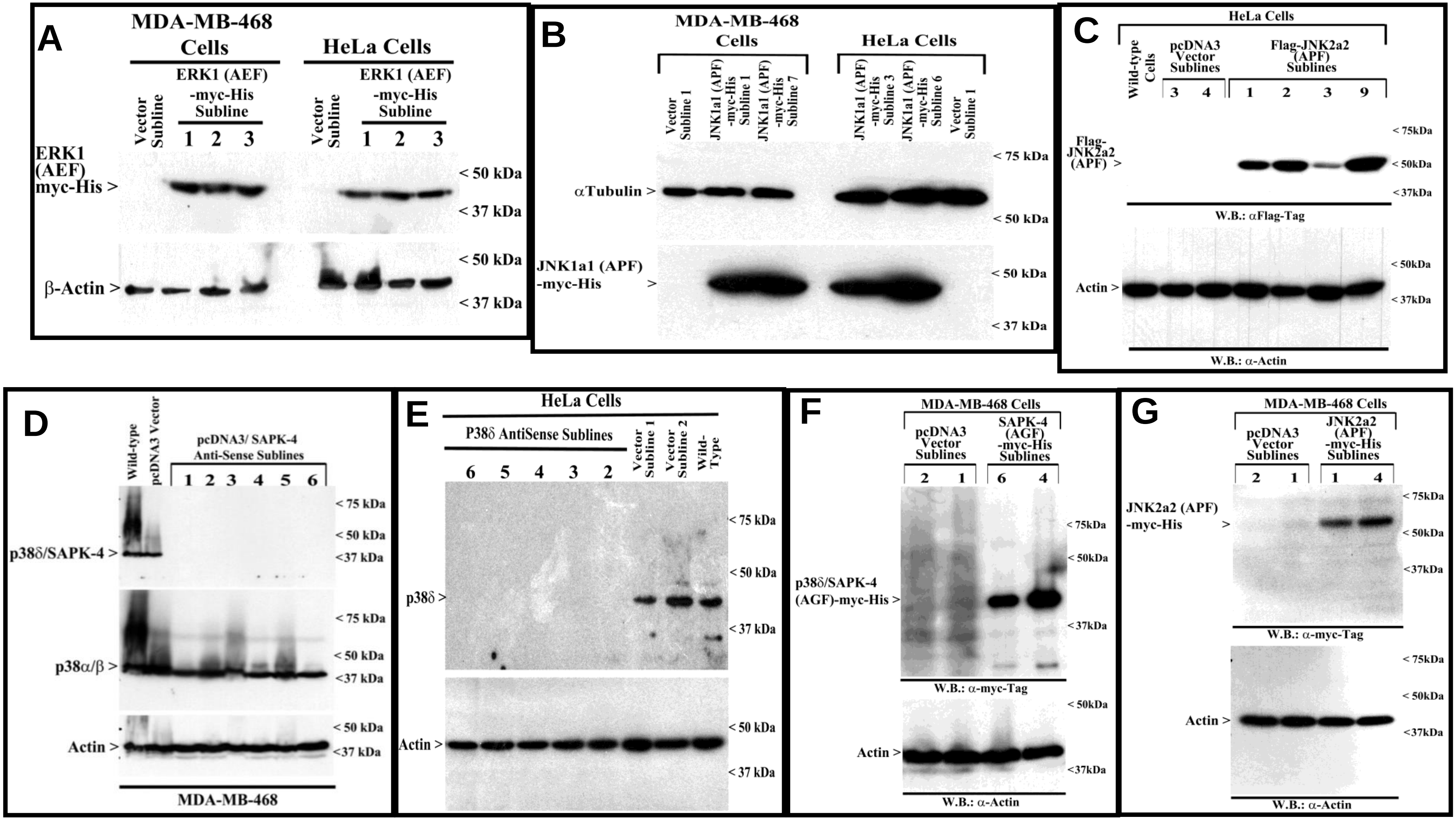
A
MDA-MB-468 Cells
HeLa Cells
ERK1 (AEF) -myc-His Subline
Vector Subline
1 2 3
ERK1 (AEF) myc-His >
β-Actin >
< 50 kDa
< 37 kDa
B
MDA-MB-468 Cells
HeLa Cells
Vector Subline 1
JNK1a1 (APF) -myc-His Subline 1
JNK1a1 (APF) -myc-His Subline 7
JNK1a1 (APF) -myc-His Subline 3
JNK1a1 (APF) -myc-His Subline 6
Vector Subline 1
αTubulin >
JNK1a1 (APF) -myc-His >
< 75 kDa
< 50 kDa
< 37 kDa
C
HeLa Cells
Wild-type Cells
pcDNA3 Vector Sublines
Flag-JNK2a2 (APF) Sublines
3 4 1 2 3 9
Flag-JNK2a2 (APF) >
W.B.: αFlag-Tag
Actin >
W.B.: α-Actin
< 75kDa
< 50kDa
< 37kDa
D
Wild-type
pcDNA3 Vector
pcDNA3/ SAPK-4 Anti-Sense Sublines
1 2 3 4 5 6
p38δ/SAPK-4 >
p38α/β >
Actin >
< 75 kDa
< 50 kDa
<37 kDa
MDA-MB-468
E
HeLa Cells
P38δ AntiSense Sublines
6 5 4 3 2
Vector Subline 1
Vector Subline 2
Wild-Type
p38δ >
Actin >
< 75 kDa
< 50 kDa
< 37 kDa
F
MDA-MB-468 Cells
pcDNA3 Vector Sublines
SAPK-4 (AGF) -myc-His Sublines
2 1 6 4
p38δ/SAPK-4 (AGF)-myc-His >
W.B.: α-myc-Tag
Actin >
W.B.: α-Actin
< 75kDa
< 50kDa
<37kDa
G
MDA-MB-468 Cells
pcDNA3 Vector Sublines
JNK2a2 (APF) -myc-His Sublines
2 1 1 4
JNK2a2 (APF) -myc-His >
W.B.: α-myc-Tag
Actin >
W.B.: α-Actin
< 75kDa
< 50kDa
<37kDa

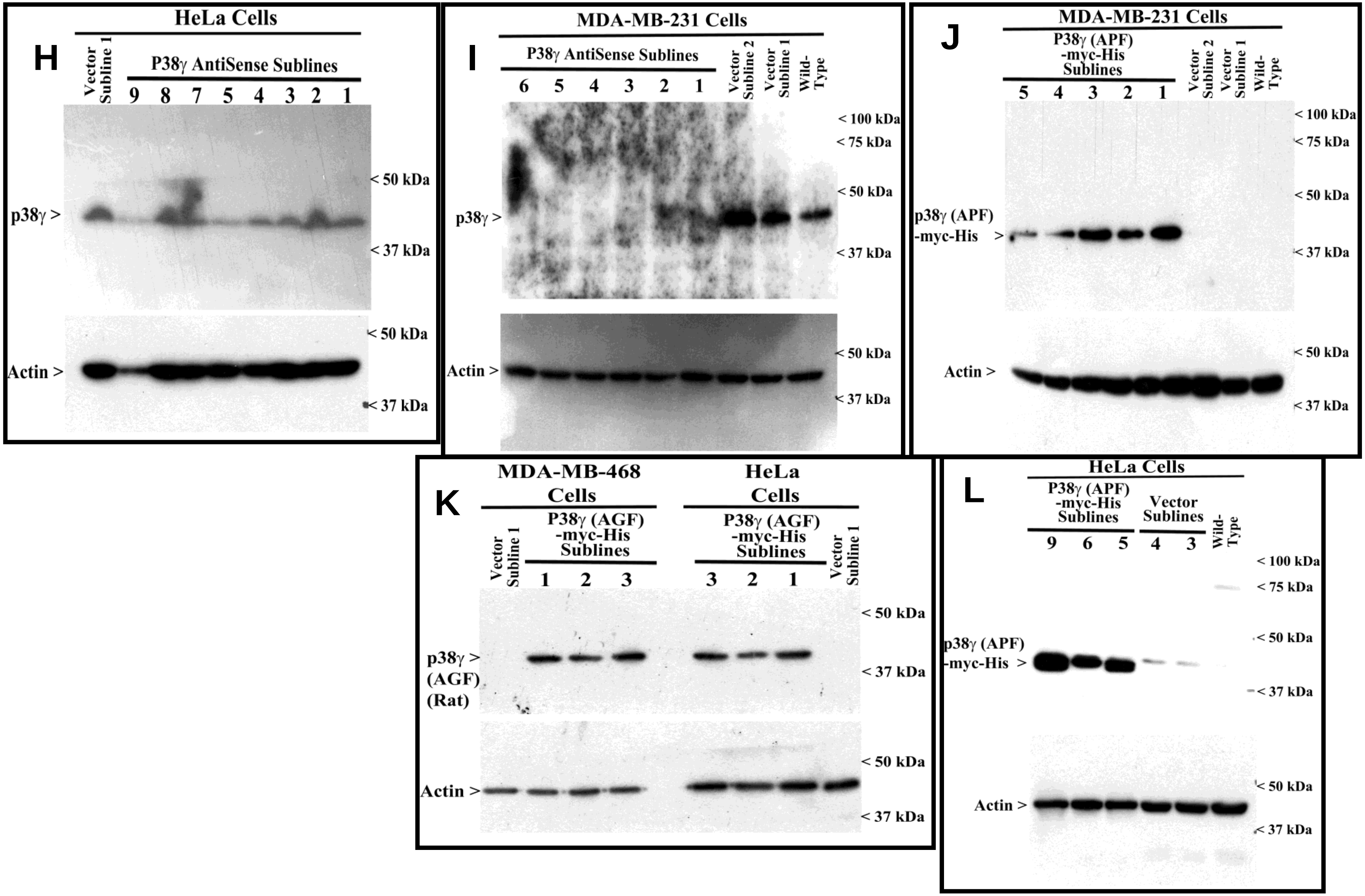

Supplementary Fig. 3: W.B. analysis of membranes containing protein lysates derived from untransfected, wild type (WT) HBC cells and stable, neomycin-resistant, HBC and HeLa sublines expressing vector, ERK1 (APF)-myc-His (A), JNK1a1 (APF) myc-His (B), JNK2a2 (APF)-myc-His (C, G), p38δ antisense sublines (D, E), p38δ/SAPK4 (AGF)-myc-His (F), p38γ antisense (H, I), P38γ (APF)-myc-His (J, L), and P38γ (AGF)-myc-His (K). Membranes were probed with myc-tag antibodies (upper blots in A, C, F, G, J, K, L; Lower blot in B), followed by anti-actin (lower blots in A, C-L) or anti-α-tubulin (upper blot in B) antibodies. In D, the membrane was also probed with anti-p38α/β antibodies (middle blot). Arrowheads on the left or right side, respectively, indicate presence of the proteins and molecular weight markers.

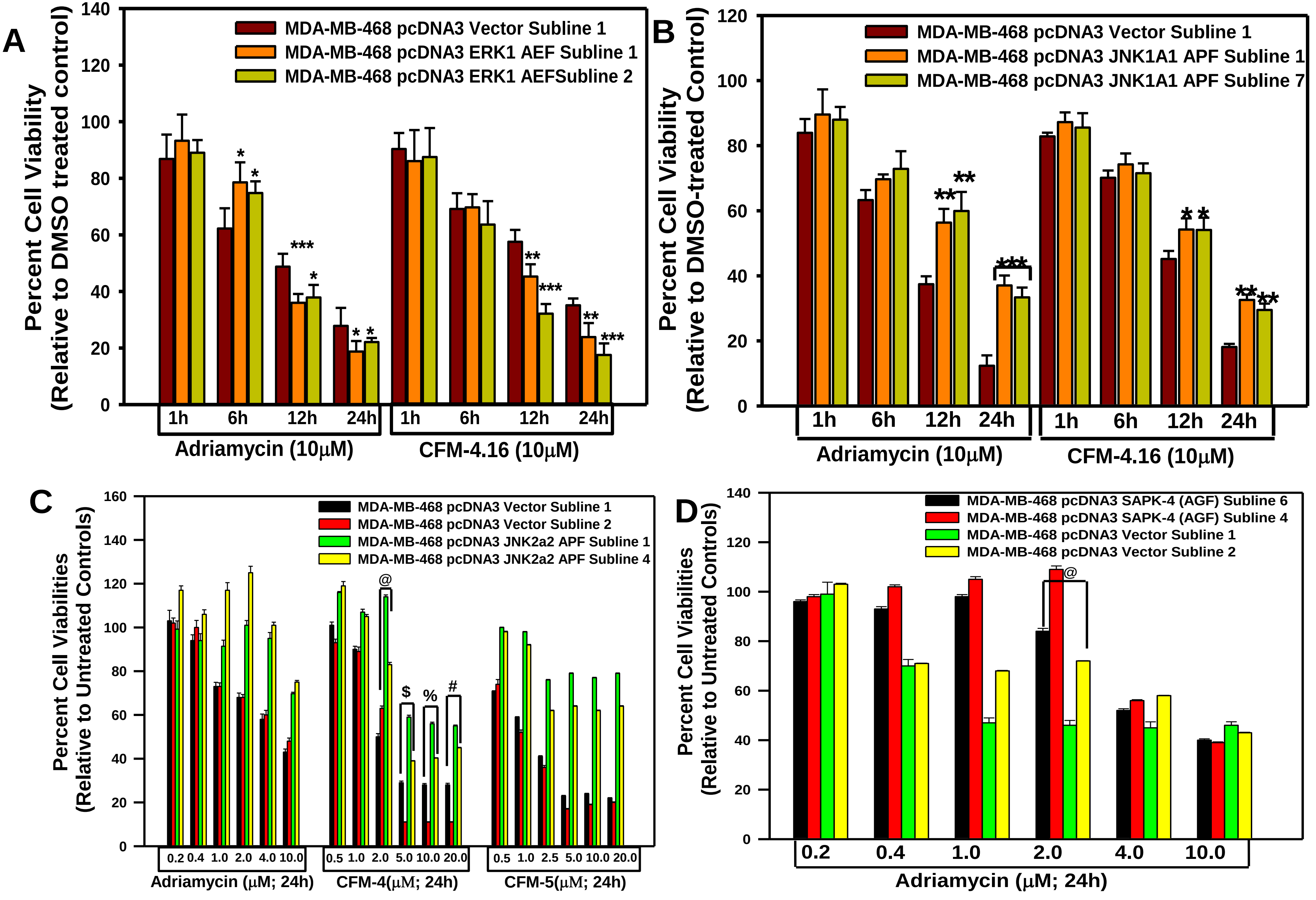
A
MDA-MB-468 pcDNA3 Vector Subline 1
MDA-MB-468 pcDNA3 ERK1 AEF Subline 1
MDA-MB-468 pcDNA3 ERK1 AEFSubline 2
Percent Cell Viability
(Relative to DMSO treated control)
1h 6h 12h 24h 1h 6h 12h 24h
Adriamycin (10μM)
CFM-4.16 (10μM)
B
MDA-MB-468 pcDNA3 Vector Subline 1
MDA-MB-468 pcDNA3 JNK1A1 APF Subline 1
MDA-MB-468 pcDNA3 JNK1A1 APF Subline 7
Percent Cell Viability
(Relative to DMSO-treated Control)
1h 6h 12h 24h 1h 6h 12h 24h
Adriamycin (10μM)
CFM-4.16 (10μM)
C
MDA-MB-468 pcDNA3 Vector Subline 1
MDA-MB-468 pcDNA3 Vector Subline 2
MDA-MB-468 pcDNA3 JNK2a2 APF Subline 1
MDA-MB-468 pcDNA3 JNK2a2 APF Subline 4
Percent Cell Viabilities
(Relative to Untreated Controls)
0.2 0.4 1.0 2.0 4.0 10.0
0.5 1.0 2.0 5.0 10.0 20.0
0.5 1.0 2.5 5.0 10.0 20.0
Adriamycin (μM; 24h)
CFM-4(μM; 24h)
CFM-5(μM; 24h)
D
MDA-MB-468 pcDNA3 SAPK-4 (AGF) Subline 6
MDA-MB-468 pcDNA3 SAPK-4 (AGF) Subline 4
MDA-MB-468 pcDNA3 Vector Subline 1
MDA-MB-468 pcDNA3 Vector Subline 2
Percent Cell Viabilities
(Relative to Untreated Controls)
0.2 0.4 1.0 2.0 4.0 10.0
Adriamycin (μM; 24h)

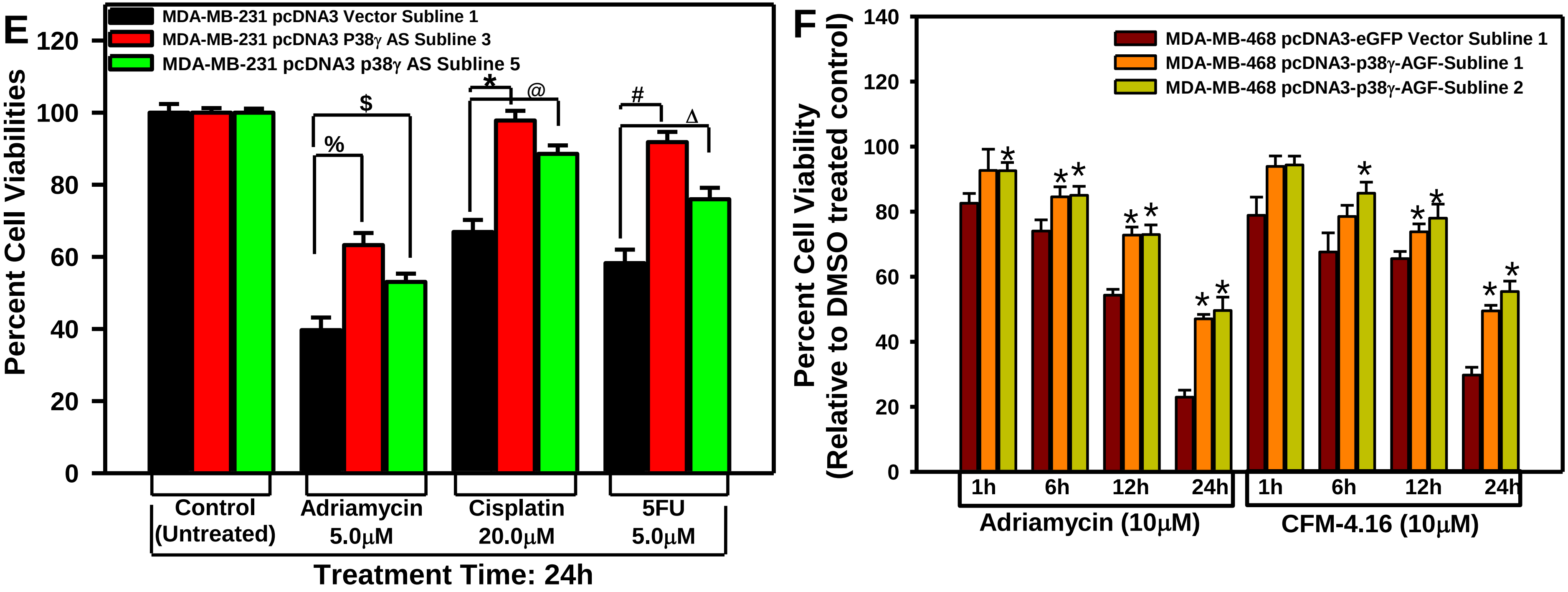

<u>*Supplementary Fig. 4:*</u> Expression of catalytically inactive MAPKs/SAPKs in HBC cells alter cell viability following treatments with DNA damaging agents. *A-F*, Cell viability was determined by MTT assay following treatments of indicated cells with vehicle/DMSO (Control) or indicated time and doses of various agents. The columns in each histogram indicate percent of live/viable cells relative to their DMSO-treated (A, B, F) or untreated (C-E) controls and represent means of three independent experiments. bars, S.E. A, B, *- p <0.05,**-p<0.01,***-p<0.001 versus the corresponding vector cells. C, D, E, @, $, %, #, *, Δ, p<0.005 versus respective vector expressing sublines. F, *-p<0.001 versus the corresponding vector cells.

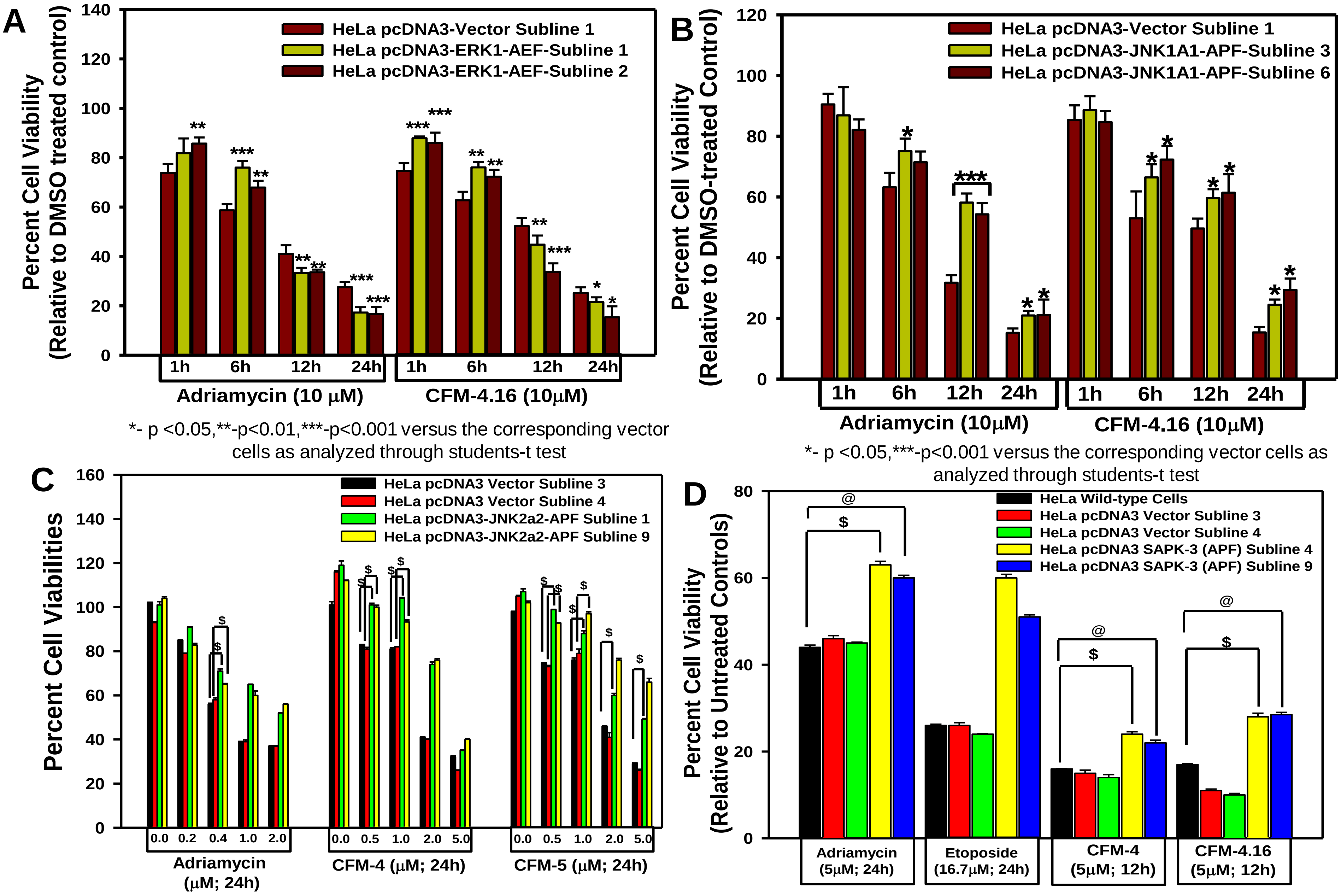
A
Percent Cell Viability (Relative to DMSO treated control)
HeLa pcDNA3-Vector Subline 1
HeLa pcDNA3-ERK1-AEF-Subline 1
HeLa pcDNA3-ERK1-AEF-Subline 2
1h 6h 12h 24h 1h 6h 12h 24h
Adriamycin (10 μM)
CFM-4.16 (10μM)
*- p <0.05,**-p<0.01,***-p<0.001 versus the corresponding vector cells as analyzed through students-t test
B
Percent Cell Viability (Relative to DMSO-treated Control)
HeLa pcDNA3-Vector Subline 1
HeLa pcDNA3-JNK1A1-APF-Subline 3
HeLa pcDNA3-JNK1A1-APF-Subline 6
1h 6h 12h 24h 1h 6h 12h 24h
Adriamycin (10μM)
CFM-4.16 (10μM)
*- p <0.05,***-p<0.001 versus the corresponding vector cells as analyzed through students-t test
C
Percent Cell Viabilities
HeLa pcDNA3 Vector Subline 3
HeLa pcDNA3 Vector Subline 4
HeLa pcDNA3-JNK2a2-APF Subline 1
HeLa pcDNA3-JNK2a2-APF Subline 9
0.0 0.2 0.4 1.0 2.0
Adriamycin (μM; 24h)
0.0 0.5 1.0 2.0 5.0
CFM-4 (μM; 24h)
0.0 0.5 1.0 2.0 5.0
CFM-5 (μM; 24h)
D
Percent Cell Viability (Relative to Untreated Controls)
HeLa Wild-type Cells
HeLa pcDNA3 Vector Subline 3
HeLa pcDNA3 Vector Subline 4
HeLa pcDNA3 SAPK-3 (APF) Subline 4
HeLa pcDNA3 SAPK-3 (APF) Subline 9
Adriamycin (5μM; 24h)
Etoposide (16.7μM; 24h)
CFM-4 (5μM; 12h)
CFM-4.16 (5μM; 12h)

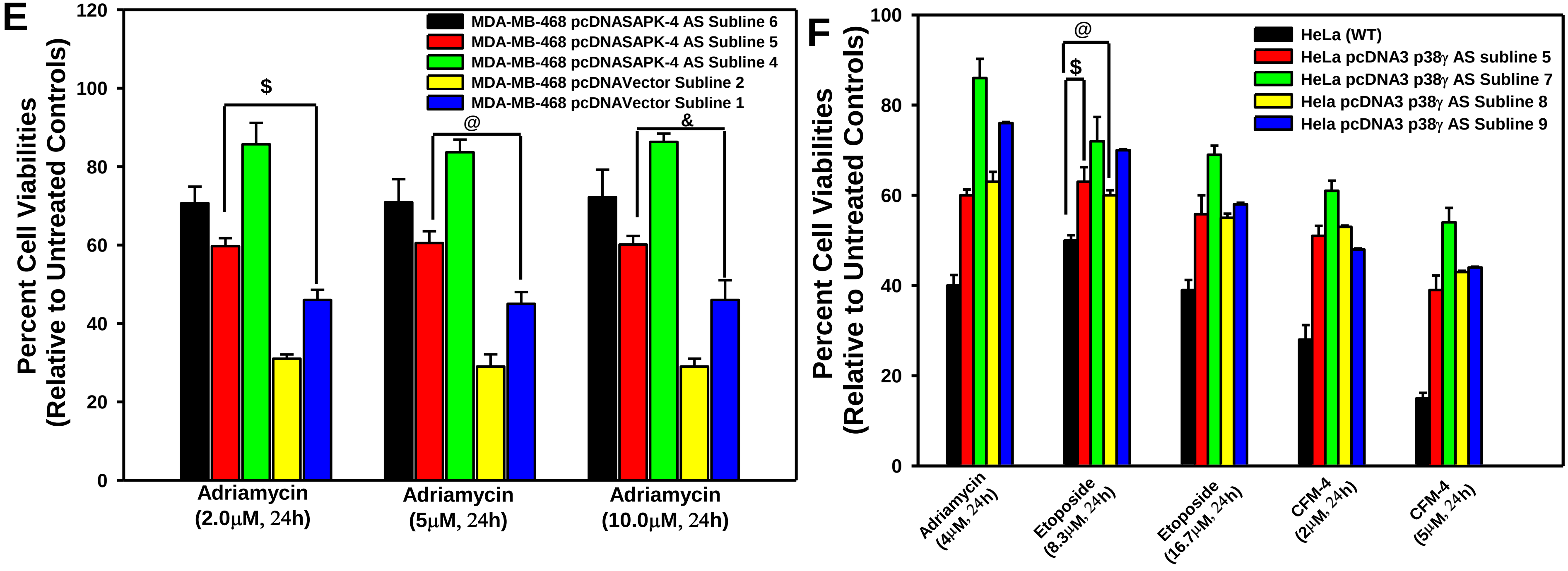

<u>*Supplementary Fig. 5:*</u> Expression of catalytically inactive MAPKs/SAPKs (A-D) or SAPK antisense (E, F) in noted cells alter cell viability following treatments with DNA damaging agents. *A-F*, Cell viability was determined by MTT assay following treatments of indicated cells with vehicle/DMSO (Control) or indicated time and doses of various agents. The columns in each histogram indicate percent of live/viable cells relative to their DMSO-treated controls (A, B) or untreated controls (C-F) and represent means of three independent experiments. bars, S.E. p <0.05,**-p<0.01,***-p<0.001 versus the corresponding vector cells (A, B), @, $, &, -p<0.01, relative to respective vector expressing sublines (C, E), @, $, -p<0.01, relative to respective wild-type cells (D, F).

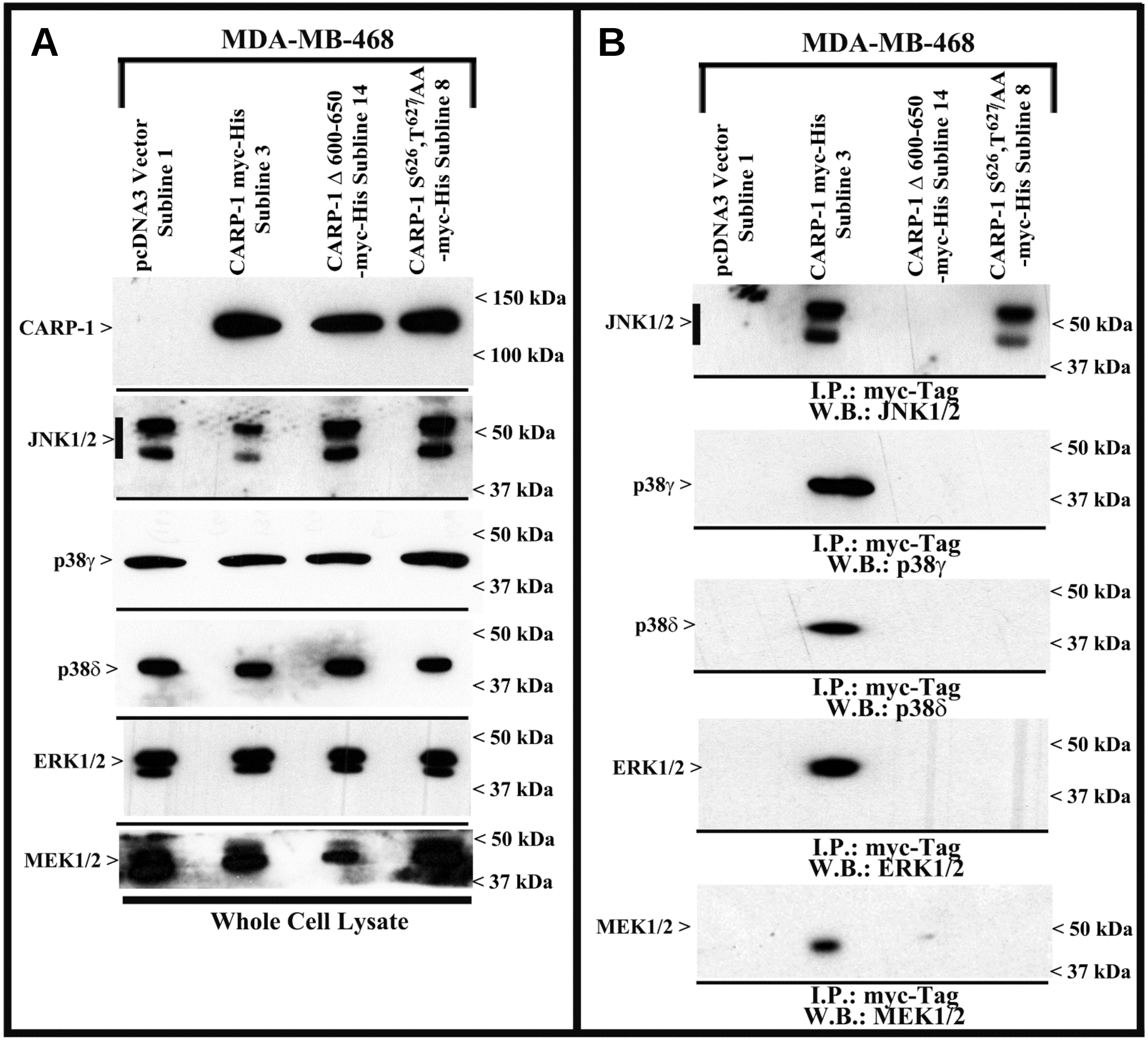
A
MDA-MB-468
pcDNA3 Vector Subline 1
CARP-1 myc-His Subline 3
CARP-1 Δ 600-650 -myc-His Subline 14
CARP-1 S626,T627/AA -myc-His Subline 8
CARP-1 >
< 150 kDa
< 100 kDa
JNK1/2 >
< 50 kDa
< 37 kDa
p38γ >
p38δ >
ERK1/2 >
MEK1/2 >
Whole Cell Lysate
B
MDA-MB-468
I.P.: myc-Tag
W.B.: JNK1/2
W.B.: p38γ
W.B.: p38δ
W.B.: ERK1/2
W.B.: MEK1/2

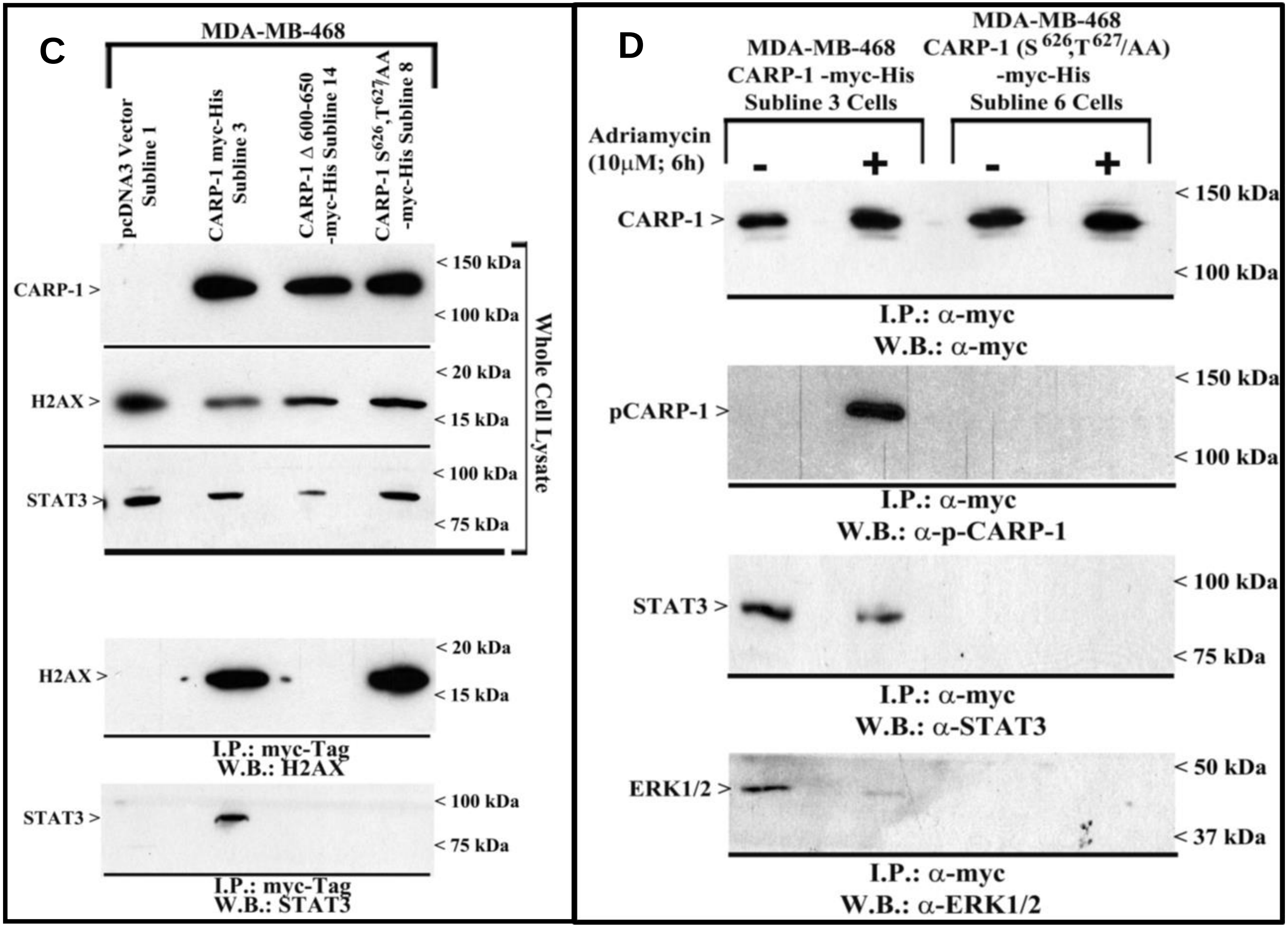
C
MDA-MB-468
pcDNA3 Vector Subline 1
CARP-1 myc-His Subline 3
CARP-1 Δ 600-650 -myc-His Subline 14
CARP-1 S626,T627/AA -myc-His Subline 8
CARP-1 >
H2AX >
STAT3 >
< 150 kDa
< 100 kDa
< 20 kDa
< 15 kDa
< 100 kDa
< 75 kDa
Whole Cell Lysate
H2AX >
< 20 kDa
< 15 kDa
I.P.: myc-Tag
W.B.: H2AX
STAT3 >
< 100 kDa
< 75 kDa
I.P.: myc-Tag
W.B.: STAT3
D
MDA-MB-468
CARP-1 -myc-His
Subline 3 Cells
MDA-MB-468
CARP-1 (S 626,T 627/AA)
-myc-His
Subline 6 Cells
Adriamycin
(10μM; 6h)
- + - +
CARP-1 >
< 150 kDa
< 100 kDa
I.P.: α-myc
W.B.: α-myc
pCARP-1 >
< 150 kDa
< 100 kDa
I.P.: α-myc
W.B.: α-p-CARP-1
STAT3 >
< 100 kDa
< 75 kDa
I.P.: α-myc
W.B.: α-STAT3
ERK1/2 >
< 50 kDa
< 37 kDa
I.P.: α-myc
W.B.: α-ERK1/2

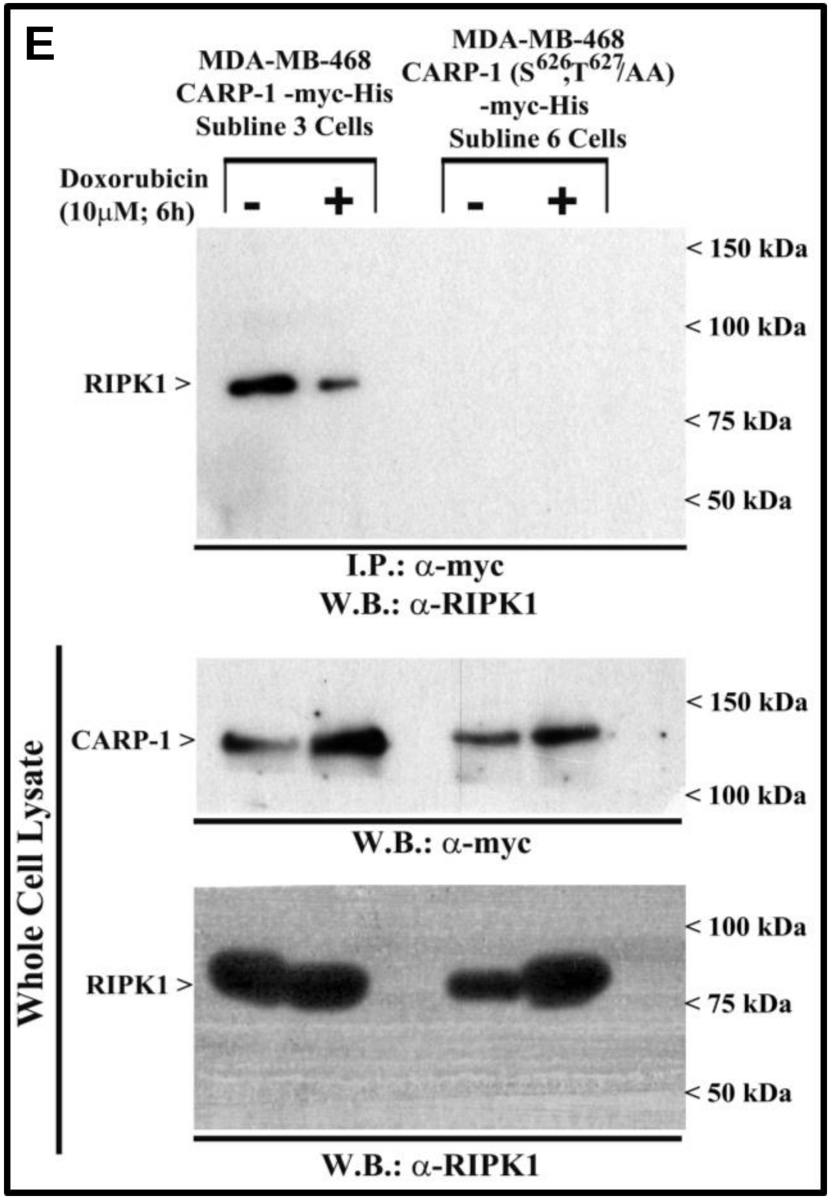

<u>*Supplementary Fig. 6:*</u> CARP-1 (WT), but not its mutants CARP-1 (Δ600-650) or CARP-1 ($S^{626}T^{627}$/AA), interact with P38γ, p38δ, ERK1/2, MEK1/2, and STAT3 (*A-C*), Adriamycin-induced CARP-1 phosphorylation interferes with CARP-1 interactions with STAT3, ERK1/2, and RIPK1 *(D, E)*. Indicated cells either untreated (-) or treated with noted dose and time of Adriamycin. In panels B-D, cell lysates were first subjected to immunoprecipitation using anti-myc tag antibodies. W.B. analysis of the immunoprecipitates or whole cell lysates was carried out using anti-p-CARP-1, anti-CARP-1 (α2), anti-JNK1/2, anti-p38γ, anti-p38δ, anti-ERK1/2, anti-MEK1/2, anti-H2AX, anti-STAT3, anti-RIPK1 antibodies. Arrowheads on the left or right side of each blot in each panel indicate presence of proteins or molecular weight markers, respectively.

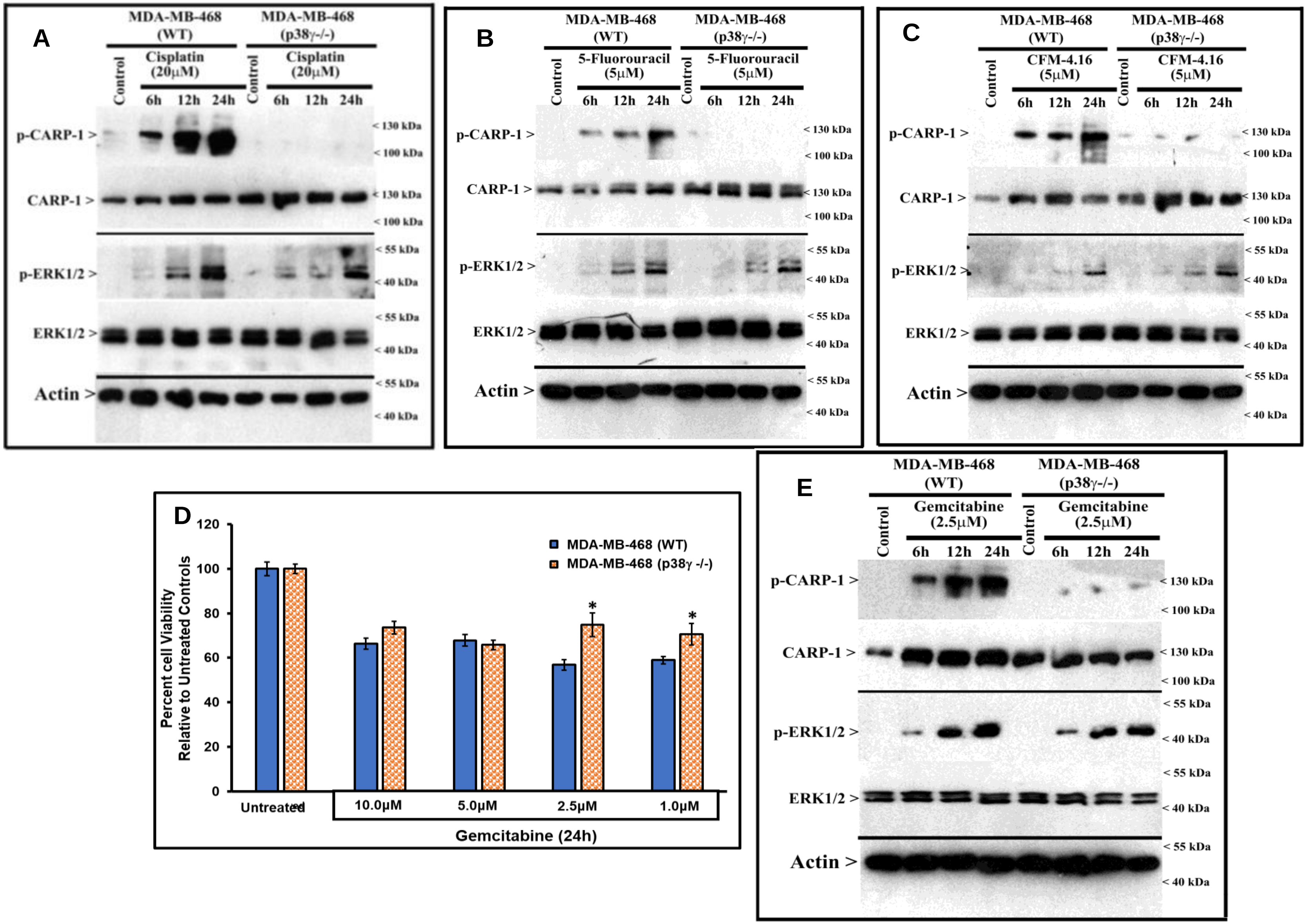

•*Supplementary Fig. 7:* DNA Damage-inducing chemotherapies robustly activate phospho-CARP-1 in wild-type HBC cells but not in cells lacking p38γ. *A-C, E*. Indicated cells were either untreated (Control) or treated with noted doses and time of respective agents. W.B. analysis of the cell lysates was carried out using anti-phospho-CARP-1, anti-CARP-1 (α2), anti-phospho-ERK1/2, anti-ERK1/2, and anti-actin antibodies. Arrowheads on the left or right side of each blot in each panel indicate presence of proteins or molecular weight markers, respectively. D. Indicated cells were either untreated or treated with noted dose and time of Gemcitabine. Cell viability was determined by MTT assay. The columns in histogram indicate percent of live/viable cells relative to their untreated controls and represent means of three independent experiments. bars, S.E. *, p<0.001 relative to corresponding WT cells.

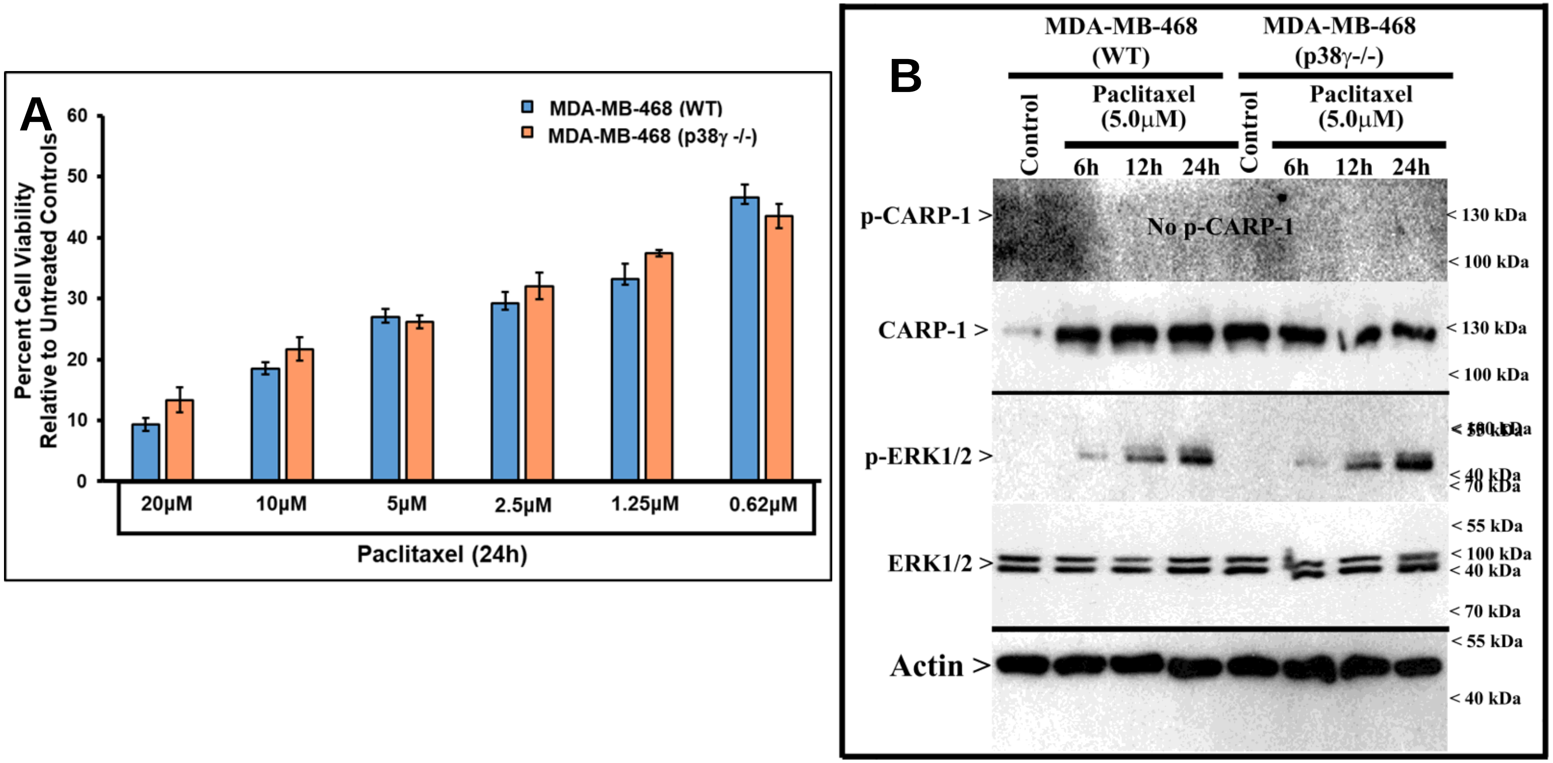

•*Supplementary Fig. 8:* Paclitaxel does not activate phospho-CARP-1 in wild-type HBC cells or in cells lacking p38γ. A, Indicated cells were either untreated or treated with noted dose and time of Gemcitabine. Cell viability was determined by MTT assay. The columns in histogram indicate percent of live/viable cells relative to their untreated controls and represent means of three independent experiments. bars, S.E. B, indicated cells were either untreated (Control) or treated with noted doses and time of paclitaxel. W.B. analysis of the cell lysates was carried out using anti-phospho-CARP-1, anti-CARP-1 (α2), anti-phospho-ERK1/2, anti-ERK1/2, and anti-actin antibodies. Arrowheads on the left or right side of each blot in each panel indicate presence of proteins or molecular weight markers, respectively.

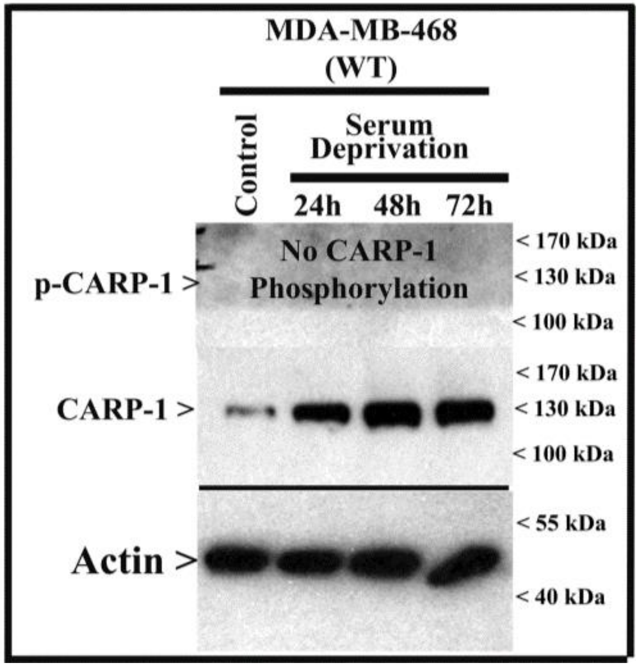

Supplementary Fig. 9: Serum deprivation does not induce CARP-1 phosphorylation. Wild-type HBC cells were either grown in normal serum conditions (Control) or grown in the absence of serum for the noted time periods. W.B. analysis of the cell lysates was carried out using anti-phospho-CARP-1, anti-CARP-1 (α2), and anti-actin antibodies. Arrowheads on the left or right side of each blot in each panel indicate presence of proteins or molecular weight markers, respectively.
