# Supplementary material for: Phosphorylation of cell cycle and apoptosis regulatory protein-1 by stress activated protein kinase P38γ is a novel mechanism of apoptosis signaling by genotoxic chemotherapy
Source: Front Oncol. 2024 May 2;14:1376666. doi: 10.3389/fonc.2024.1376666 (PMC11096501; doi:10.3389/fonc.2024.1376666)
Supplement: Supplementary file 2 [file Table_1.docx]

| **Table S1** | | | | |
| --- | --- | --- | --- | --- |
| **Antibody** | **Source** | **Type** | **Manufacturer** | **Product number** |
| GST | Rabbit | mAb | Cell Signaling Technology | 2625 |
| myc | Mouse | mAb | Cell Signaling Technology | 2276 |
| P38 γ | Rabbit | pAb | Cell Signaling Technology | 2307 |
| p38 δ | Rabbit | mAb | Cell Signaling Technology | 2308 |
| P38α/β | Rabbit | mAb | Cell Signaling Technology | 8690 |
| p-P38 α/β | Rabbit | pAb | Cell Signaling Technology | 9211 |
| RIPK1 | Rabbit | mAb | Cell Signaling Technology | 3493 |
| STAT3 | Rabbit | mAb | Cell Signaling Technology | 4904 |
| P-STAT3 (S727) | Rabbit | pAb | Cell Signaling Technology | 9134 |
| P-STAT3 (SY705) | Rabbit | mAb | Cell Signaling Technology | 9145 |
| JNK1/2 | Rabbit | mAb | Cell Signaling Technology | 9258 |
| P-JNK1/2 | Mouse | mAb | Cell Signaling Technology | 9255 |
| MKK4 | Rabbit | pAb | Cell Signaling Technology | 9152 |
| p-MKK4 | Rabbit | mAb | Cell Signaling Technology | 4514 |
| ERK1 | Mouse | mAb | Cell Signaling Technology | 4696 |
| P-ERK1 | Mouse | mAb | Cell Signaling Technology | 5726 |
| P65 | Rabbit | mAb | Cell Signaling Technology | 8242 |
| p-P65 | Mouse | mAb | Cell Signaling Technology | 13346 |
| MEK1/2 | Rabbit | mAb | Cell Signaling Technology | 9126 |
| CDK4 | Rabbit | mAb | Cell Signaling Technology | 12790 |
| CDK6 | Rabbit | mAb | Cell Signaling Technology | 13331 |
| H2AX | Rabbit | mAb | Cell Signaling Technology | 76631 |
| Cleaved PARP1 | Mouse | mAb | Cell Signaling Technology | 9546 |
| p-CARP-1 (T^627^) | Rabbit | pAb | Custom Synthesis by Eurogentec, Seraing, Belgium | This Report |

***Table S1:* List of various antibodies that were utilized in this report.**
