# Supplementary material for: Phosphorylation of cell cycle and apoptosis regulatory protein-1 by stress activated protein kinase P38γ is a novel mechanism of apoptosis signaling by genotoxic chemotherapy
Source: Front Oncol. 2024 May 2;14:1376666. doi: 10.3389/fonc.2024.1376666 (PMC11096501; doi:10.3389/fonc.2024.1376666)
Supplement: Supplementary file 3 [file Table_2.docx]

| **Table S2** | | | |
| --- | --- | --- | --- |
| **Recombinant Plasmid** | **Vector** | **Resistance Marker(s)** | **Reference(s)** |
| **pcDNA3/Vector** | **pcDNA3/Vector** | **Neomycin/Ampicillin** | **Ref.9** |
| **pcDNA3/eGFP Vector** | **pcDNA3/Vector** | **Neomycin/Ampicillin** | **This Report** |
| **pcDNA3/CARP-1 (WT)-myc-His** | **pcDNA3/Vector** | **Neomycin/Ampicillin** | **Ref.9** |
| **pcDNA3/CARP-1 (S^626^,T^627^, T^629^/AAA)-myc-His** | **pcDNA3/Vector** | **Neomycin/Ampicillin** | **This Report** |
| **pcDNA3/CARP-1 (S^626^,T^627^/AA)-myc-His** | **pcDNA3/Vector** | **Neomycin/Ampicillin** | **This Report** |
| **pcDNA3/CARP-1 Δ600-650-myc-His** | **pcDNA3/Vector** | **Neomycin/Ampicillin** | **This Report** |
| **pcDNA3/CARP-1 Δ637-667-myc-His** | **pcDNA3/Vector** | **Neomycin/Ampicillin** | **Ref.10** |
| **pcDNA3/ERK1(AEF)-myc-His** | **pcDNA3/Vector** | **Neomycin/Ampicillin** | **This Report** |
| **pcDNA3/JNK1a1(APF)-myc-His** | **pcDNA3/Vector** | **Neomycin/Ampicillin** | **This Report** |
| **pcDNA3/JNK2a2(APF)-myc-His** | **pcDNA3/Vector** | **Neomycin/Ampicillin** | **This Report** |
| **pcDNA3/p38δ/SAPK4 AS (Antisense)** | **pcDNA3/Vector** | **Neomycin/Ampicillin** | **This Report** |
| **pcDNA3/p38δ/SAPK4(AGF)-myc-His** | **pcDNA3/Vector** | **Neomycin/Ampicillin** | **This Report** |
| **pcDNA3/P38γ AS(Antisense)** | **pcDNA3/Vector** | **Neomycin/Ampicillin** | **This Report** |
| **pcDNA3-P38γ(AGF)-myc-His** | **pcDNA3/Vector** | **Neomycin/Ampicillin** | **This Report** |
| **pcDNA3/SAPK3/P38γ(APF)-myc-His** | **pcDNA3/Vector** | **Neomycin/Ampicillin** | **This Report** |
| **pcDNA3-EGFP-CARP-1 (611-640)** | **pcDNA3/Vector** | **Neomycin/Ampicillin** | **This Report** |
| **pcDNA3/Flag JNK1a1** | **pcDNA3 Vector** | **Neomycin/Ampicillin** | **Addgene^1^** |
| **pcDNA3/Flag JNK1a1 (APF)** | **pcDNA3 Vector** | **Neomycin/Ampicillin** | **Addgene^2^** |
| **pcDNA3/Flag JNK2a2 (APF)** | **pcDNA3 Vector** | **Neomycin/Ampicillin** | **Addgene^3^** |
| **pcDNA3/Flag p38γ (AGF)** | **pcDNA3 Vector** | **Neomycin/Ampicillin** | **Addgene^4^** |

***Table S2:* List of various recombinant plasmids that were either obtained from Addgene or generated in this report.**

**Addgene Plasmid Refs:**

**^1^#13798 and ^2^#13846: JNK1: a protein kinase stimulated by UV light and Ha-Ras that binds and phosphorylates the c-Jun activation domain. Derijard B, Hibi M, Wu IH, Barrett T, Su B, Deng T, Karin M, Davis RJ. Cell. 1994 Mar 25. 76(6):1025-37. 10.1016/0092-8674(94)90380-8.**

**^3^#13761: Gupta S, Barrett T, Whitmarsh AJ, Cavanagh J, Sluss HK, Derijard B, Davis RJ. EMBO J. 1996 Jun 3. 15(11):2760-70.**
