# Supplementary material for: Phosphorylation of cell cycle and apoptosis regulatory protein-1 by stress activated protein kinase P38γ is a novel mechanism of apoptosis signaling by genotoxic chemotherapy
Source: Front Oncol. 2024 May 2;14:1376666. doi: 10.3389/fonc.2024.1376666 (PMC11096501; doi:10.3389/fonc.2024.1376666)
Supplement: Supplementary file 4 [file Table_3.docx]

| **Table S3** | | |
| --- | --- | --- |
| **Cell Line** | **Source** | **Reference(s)** |
| **MDA-MB-468 Breast Cancer** | **ATCC** | **Ref. 8, 9, 10, 11** |
| **MDA-MB-231 Breast Cancer** | **ATCC** | **Ref. 8, 9, 10, 11** |
| **HeLa Cervical Cancer** | **ATCC** | **Ref 8, 11** |
| **A498 Renal Cancer** | **ATCC** | **Ref. 13** |
| **UOK 262 Renal Cancer** | **Dr. Marsten Lanehan, NCI** | **Ref. 13** |
| **HK2 Renal Epithelial Cells** | **ATCC** | **Ref. 13** |
| **MDA-MB-468 (P38γ-/-)** | **Biocytogen, Inc** | **This Report** |
| **HL1 Cardiomyocytes** | **Dr. Karin Przyklenk** | **Ref. 11** |
| **4T1 Mouse Breast Cancer** | **Dr. Lisa Polin, Karmanos Cancer Institute, Detroit** | **This Report, Ref. 8** |
| **Mouse Embryonic Fibroblasts (WT)** | **Dr. Anna Cuenda, Spain** | **Ref. 26** |
| **Mouse Embryonic Fibroblasts (p38γ-/-)** | **Dr. Anna Cuenda, Spain** | **Ref. 26** |
| **Human Mammary Epithelial Cells (HMEC)** | **Lonza, Walkersville, MD** | **This Report** |
| **C2C12 Myoblast cell Line** | **Dr. Miriam Greenberg, Department of Biological Sciences, Wayne State University, Detroit, MI** | **This Report, Ref. 32** |
| **HC11 mouse breast epithelial cells** | **Dr. Laura L. Hernandez, Department of Animal and Dairy Sciences, University of Wisconsin-Madison, WI** | **This Report, Ref. 33** |
| **W0069 BRCA-deficient mouse mammary tumor cells** | **Dr. Karen Liby, Department of Pharmacology and Toxicology, Michigan State University, East Lansing, MI 48824** | **This Report, Ref. 34** |

***Table S3:* List of various cell lines and the source from where they were obtained.**
