# Supplementary material for: Phosphorylation of cell cycle and apoptosis regulatory protein-1 by stress activated protein kinase P38γ is a novel mechanism of apoptosis signaling by genotoxic chemotherapy
Source: Front Oncol. 2024 May 2;14:1376666. doi: 10.3389/fonc.2024.1376666 (PMC11096501; doi:10.3389/fonc.2024.1376666)
Supplement: Supplementary file 5 [file Table_4.docx]

| **Table** S4 | | |
| --- | --- | --- |
| **Stable Sublines** | **Resistance Marker(s)** | **Reference(s)** |
| **MDA-MB-468-pcDNA3/Vector** | **Neomycin** | **Ref.9** |
| **MDA-MB-468-pcDNA3/eGFP Vector** | **Neomycin** | **This Report** |
| **MDA-MB-468-pcDNA3/CARP-1 (WT)-myc-His** | **Neomycin** | **Ref.9** |
| **MDA-MB-468-pcDNA3/CARP-1 (S^626^,T^627^, T^629^/AAA)-myc-His** | **Neomycin** | **This Report** |
| **MDA-MB-468-pcDNA3/CARP-1 (S^626^,T^627^/AA)-myc-His** | **Neomycin** | **This Report** |
| **MDA-MB-468-pcDNA3/CARP-1 Δ600-650-myc-His** | **Neomycin** | **This Report** |
| **MDA-MB-468-pcDNA3/CARP-1 Δ637-667-myc-His** | **Neomycin** | **Ref.10** |
| **MDA-MB-468-pcDNA3/ERK1(AEF)-myc-His** | **Neomycin** | **This Report** |
| **HeLa-pcDNA3/ERK1(AEF)-myc-His** | **Neomycin** | **This Report** |
| **MDA-MB-468-pcDNA3/JNK1a1(APF)-myc-His** | **Neomycin** | **This Report** |
| **MDA-MB-468-pcDNA3/JNK1a1(APF)-myc-His** | **Neomycin** | **This Report** |
| **MDA-MB-468-pcDNA3/JNK2a2(APF)-myc-His** | **Neomycin** | **This Report** |
| **HeLa-pcDNA3/JNK2a2(APF)-myc-His** | **Neomycin** | **This Report** |
| **MDA-MB-468-pcDNA3/p38δ/SAPK4 AS (Antisense)** | **Neomycin** | **This Report** |
| **HeLa-pcDNA3/p38δ/SAPK4 AS (Antisense)** | **Neomycin** | **This Report** |
| **MDA-MB-468-pcDNA3/p38δ/SAPK4(AGF)-myc-His** | **Neomycin** | **This Report** |
| **HeLa-pcDNA3/P38γ AS(Antisense)** | **Neomycin** | **This Report** |
| **MDA-MB-231-pcDNA3/P38γ AS(Antisense)** | **Neomycin** | **This Report** |
| **MDA-MB-468-pcDNA3-P38γ(AGF)-myc-His** | **Neomycin** | **This Report** |
| **HeLa-pcDNA3-P38γ(AGF)-myc-His** | **Neomycin** | **This Report** |
| **MDA-MB-231-pcDNA3/SAPK3/P38γ(APF)-myc-His** | **Neomycin** | **This Report** |
| **HeLa-pcDNA3/SAPK3/P38γ(APF)-myc-His** | **Neomycin** | **This Report** |
| **MDA-MB-468-pcDNA3-EGFP-CARP-1 (611-640)** | **Neomycin** | **This Report** |
| **HeLa-pcDNA3-EGFP-CARP-1 (611-640)** | **Neomycin** | **This Report** |

***Table S4:* List of various plasmid-transfected stable cell lines.**
