# Supplementary material for: Phosphorylation of cell cycle and apoptosis regulatory protein-1 by stress activated protein kinase P38γ is a novel mechanism of apoptosis signaling by genotoxic chemotherapy
Source: Front Oncol. 2024 May 2;14:1376666. doi: 10.3389/fonc.2024.1376666 (PMC11096501; doi:10.3389/fonc.2024.1376666)
Supplement: Supplementary file 8 [file Table_7.docx]

| **Table S7** | | | |
| --- | --- | --- | --- |
| **Target Kinase** | **Percent Phosphorylation of CARP-1 Peptide** | | |
|  | **Wild-type** | **S^626^T^627^/AA** | **S^626^T^627^T^629^/AAA** |
| **AuroraA** | **3** | **3** | **3** |
| **AuroraB** | **0** | **1** | **1** |
| **DAPK3** | **3** | **3** | **4** |
| **NIK** | **12** | **11** | **13** |
| **STK3** | **15** | **14** | **13** |
| **TGFβR2** | **19** | **15** | **16** |
| **CDK4/CyclinD1** | **16** | **12** | **12** |
| **CDK6/CyclinD3** | **17** | **15** | **16** |
| **IKKα** | **6** | **5** | **4** |
| **IKKβ** | **2** | **1** | **1** |
| **PLK1** | **12** | **12** | **11** |
| **MLCK** | **1** | **0** | **0** |
| **CDK1/CyclinB1** | **9** | **1** | **2** |
| **CDK1/CyclinA1** | **14** | **1** | **1** |
| **CDK2/CyclinA1** | **2** | **0** | **0** |
| **CDK3/CyclinE1** | **3** | **1** | **11** |
| **PIM1** | **0** | **0** | **1** |
| **MEK2** | **2** | **0** | **0** |
| **PKAcα** | **0** | **0** | **0** |
| **PKCα** | **10** | **9** | **9** |
| **ABL1** | **2** | **2** | **2** |
| **AKT1** | **1** | **0** | **0** |
| **SGK2** | **0** | **0** | **0** |
| **SGK3** | **0** | **0** | **0** |
| **ERK1** | **34** | **0** | **0** |
| **HIPK2** | **5** | **1** | **1** |
| **TAIK1** | **11** | **8** | **7** |
| **RSK1** | **0** | **0** | **0** |
| **DAPK1** | **1** | **1** | **1** |
| **JNK1** | **12** | **3** | **3** |
| **AMPK(A1/B1/G1)** | **5** | **4** | **5** |
| **DAPK2** | **1** | **1** | **2** |
| **CK1α1** | **3** | **3** | **3** |
| **GSK3β** | **1** | **0** | **0** |
| **MAPKAP2** | **1** | **1** | **1** |
| **p38α** | **4** | **0** | **0** |
| **p70S6K** | **1** | **1** | **2** |
| **CDK7/CyclinH** | **19** | **21** | **18** |
| **CHK1** | **3** | **3** | **4** |
| **CHK2** | **0** | **0** | **0** |
| **RIPK1** | **1** | **1** | **0** |
| **RIPK3** | **4** | **4** | **4** |

Wild-type peptide: EQDEEEKDDGEAKEISTPTHWSKLDPKTMK;

S^626^T^627^/AA peptide: EQDEEEKDDGEAKEIAAPTHWSKLDPKTMK;

S^626^T^627^T^629^/AAA peptide: EQDEEEKDDGEAKEIAAPAHWSKLDPKTMK

***Table S7:* In vitro phosphorylation of CARP-1 peptides by various kinases.**
