# Supplementary material for: Phosphorylation of cell cycle and apoptosis regulatory protein-1 by stress activated protein kinase P38γ is a novel mechanism of apoptosis signaling by genotoxic chemotherapy
Source: Front Oncol. 2024 May 2;14:1376666. doi: 10.3389/fonc.2024.1376666 (PMC11096501; doi:10.3389/fonc.2024.1376666)
Supplement: Supplementary file 9 [file Table_8.docx]

|  |  | **Total number of cases (n=504)** |
| --- | --- | --- |
| **p-CARP1** | Positive | 83 |
|  | Negative | 421 |
| **Estrogen Receptor** | Positive | 367 |
|  | Negative | 111 |
|  | Unknown | 26 |
| **Progesterone Receptor** | Positive | 297 |
|  | Negative | 164 |
|  | Unknown | 43 |
| **Radiation therapy** | Yes | 325 |
|  | No | 162 |
|  | Unknown | 17 |
| **Neoadjuvant chemotherapy** | Yes | 37 |
|  | No | 452 |
|  | Unknown | 15 |
| **Endocrine therapy** | Yes | 298 |
|  | No | 186 |
|  | Unknown | 20 |
| **Local recurrence** | Yes | 85 |
|  | No | 407 |
|  | Unknown | 12 |
| **Metastasis** | Yes | 145 |
|  | No | 344 |
|  | Unknown | 15 |

***Table S8:* Analysis of breast cancer tumor microarrays for expression of phospho-CARP-1. The set of TMA slides was stained with anti-phospho-CARP-1 as well as anti-CARP-1 (α2) antibodies as detailed in Methods The table also indicates tumors numbers with hormone status, prior treatment, metastasis, and recurrence parameters.**
